# Heterogeneity of the Axon Initial Segment in Interneurons and Pyramidal Cells of Rodent Visual Cortex

**DOI:** 10.3389/fncel.2017.00332

**Published:** 2017-11-06

**Authors:** Felix Höfflin, Alexander Jack, Christian Riedel, Julia Mack-Bucher, Johannes Roos, Corinna Corcelli, Christian Schultz, Petra Wahle, Maren Engelhardt

**Affiliations:** ^1^Institute of Neuroanatomy, Medical Faculty Mannheim, Center for Biomedicine and Medical Technology Mannheim (CBTM), Heidelberg University, Heidelberg, Germany; ^2^Developmental Neurobiology, Department of Zoology and Neurobiology, Ruhr-University Bochum, Bochum, Germany; ^3^Live Cell Imaging Core Mannheim (LIMA), Medical Faculty Mannheim, Center for Biomedicine and Medical Technology Mannheim (CBTM), Heidelberg University, Heidelberg, Germany

**Keywords:** axon initial segment, interneuron, pyramidal neuron, βIV-spectrin, axon-carrying dendrite cell, basket cell

## Abstract

The microdomain that orchestrates action potential initiation in neurons is the axon initial segment (AIS). It has long been considered to be a rather homogeneous domain at the very proximal axon hillock with relatively stable length, particularly in cortical pyramidal cells. However, studies in other brain regions paint a different picture. In hippocampal CA1, up to 50% of axons emerge from basal dendrites. Further, in about 30% of thick-tufted layer V pyramidal neurons in rat somatosensory cortex, axons have a dendritic origin. Consequently, the AIS is separated from the soma. Recent *in vitro* and *in vivo* studies have shown that cellular excitability is a function of AIS length/position and somatodendritic morphology, undermining a potentially significant impact of AIS heterogeneity for neuronal function. We therefore investigated neocortical axon morphology and AIS composition, hypothesizing that the initial observation of seemingly homogeneous AIS is inadequate and needs to take into account neuronal cell types. Here, we biolistically transfected cortical neurons in organotypic cultures to visualize the entire neuron and classify cell types in combination with immunolabeling against AIS markers. Using confocal microscopy and morphometric analysis, we investigated axon origin, AIS position, length, diameter as well as distance to the soma. We find a substantial AIS heterogeneity in visual cortical neurons, classified into three groups: (I) axons with somatic origin with proximal AIS at the axon hillock; (II) axons with somatic origin with distal AIS, with a discernible gap between the AIS and the soma; and (III) axons with dendritic origin (axon-carrying dendrite cell, AcD cell) and an AIS either starting directly at the axon origin or more distal to that point. Pyramidal cells have significantly longer AIS than interneurons. Interneurons with vertical columnar axonal projections have significantly more distal AIS locations than all other cells with their prevailing phenotype as an AcD cell. In contrast, neurons with perisomatic terminations display most often an axon originating from the soma. Our data contribute to the emerging understanding that AIS morphology is highly variable, and potentially a function of the cell type.

## Introduction

A common assumption is that the axon initial segment (AIS) in most cortical neurons spans the very proximal portion of the axon that emerges from the soma at the axon hillock. This position is of importance for its general function: the initiation, propagation, and backpropagation of action potentials (APs). Consequently, the AIS is characterized by a high density clustering of voltage-gated sodium and potassium channels (Kole and Stuart, 2012), rendering it the ideal site for AP initiation in a neuron (Debanne et al., 2011). These channels are tethered in the axonal membrane by specific membrane scaffolding proteins, particularly ankyrin G and its binding partner βIV-spectrin, which then connect to the axonal cytoskeleton, building a stable, periodic structure (Rasband, 2010; D’Este et al., 2015; Leterrier, 2016).

Axon onset and AIS emergence at neurons is often described as generally proximal to the soma (Rasband, 2010; Grubb et al., 2011). However, different axon onsets in various neuron classes have been described previously, going back as far as the works of Ramón y Cajal (Cajal, 1909). More recently, several studies pointed out varying degrees of heterogeneity in axon onset, e.g., in dopaminergic neurons of the rodent substantia nigra (Häusser et al., 1995; Gentet and Williams, 2007), hippocampal oriens-alveus interneurons (Martina et al., 2000), and hypothalamic neuroendocrine neurons (Herde et al., 2013). In cortex, the current knowledge is incomplete. Subpopulations of cortical principal neurons in both primate (Sloper and Powell, 1979) and cat (Peters et al., 1968) have been shown to harbor axons off a dendrite (for review see Triarhou, 2014). Lorincz and Nusser (2010) showed that in CA1 pyramidal neurons, the apical dendrite can serve as origin for the axon. In roughly 50% of CA1 pyramidal cells, the axon arises from a basal dendrite, and Thome et al. (2014) coined the term “axon-carrying dendrite cell”, AcD cell. Interneurons of different types often carry axons on dendrites, and some even have more than one axon (Meyer and Wahle, 1988). Also, bipolar cells give rise to vertically projecting axons mainly from a more substantial and longer descending dendrite (Peters and Kimerer, 1981; Meyer, 1983). The dendritic origin of axons and hence their AIS has functional implications. For example, in hippocampus CA1, AcD cells show higher excitability to synaptic input and generate APs with lower activation thresholds (Thome et al., 2014). A similar phenomenon was recently observed after electrophysiological recording and modeling of mouse cerebellar granule cells (Houston et al., 2017). Further, about 1/3 of all evaluated thick-tufted pyramidal cells in somatosensory cortex layer V give rise to axons from dendrites and are further characterized by a reduced dendritic complexity and thinner main apical dendrites (Hamada et al., 2016). The authors provide evidence that the AIS location in these cells is correlated with the somatodendritic capacitance load, and suggest it to be a mechanism for homeostatic scaling of somatic APs.

Several studies have shown that the AIS is not a static microdomain. Rather, its structural and functional plasticity is believed to contribute to homeostatic mechanisms and neuronal function (reviewed in Yamada and Kuba, 2016). A recent study applying computational modeling of realistic neuronal cell types indicated that intrinsic excitability correlates with the somatodendritic domain that corresponds to a certain AIS location and length (Gulledge and Bravo, 2016). Further, in the nucleus laminaris of the avian auditory system, AIS length and location varies according to functional cell specialization: Neurons responding to high-frequency sounds have short AIS that are located distally on the axon. In contrast, neurons processing low-frequency sounds tend to have longer and more proximal AIS (Kuba et al., 2006). Also, a significant body of evidence from *in vitro* studies underlines our understanding of the AIS as a dynamically regulated, adaptive microdomain with the potential to regulate cellular input-output relationships and thus impact neuronal network state (reviewed in Wefelmeyer et al., 2016; Jamann et al., 2017).

Assuming that AIS morphology correlates with cellular function and that neurons utilize AIS plasticity to regulate excitability, we hypothesize that AIS length and location have to be significantly more heterogeneous in sensory cortex than it is currently acknowledged. In addition, current data on AIS length and position particularly in interneurons is limited. Therefore, we set out to investigate AIS morphology, first characterizing three distinct axon morphologies *in vivo*. We then used biolistic gene transfection in organotypic cultures derived from postnatal rat visual cortex to analyze these morphological classes in more detail. Our data indicate that three major morphological groups exist into which pyramidal neurons and interneurons can be separated. Analyzing AIS length, we find that pyramidal neurons in total have significantly longer AIS than interneurons. Interestingly, interneurons with vertical columnar axonal projections display significantly more distal AIS locations than all other cells in that their prevailing phenotype is that of an AcD cell.

## Materials and Methods

All antibodies used in this study (*in vivo*, *ex vivo*, *in vitro*) are summarized in Table 1.

**Table 1:** Specification of antibodies used in the study with indication of catalog number, clone, working dilution, sources and references or research resource identifiers (RRID) where available.

Antibody (species), Catalog No., Clone/type	Dil.	Source	RRID or Reference
Ankyring (rb), sc-28561, H-215	1:500	UC Davis/NIH NeuroMab Facility, CA, USA	AB_633909
ßIV-spectrin (rb)	1:500	Selfmade, directed against amino acids 2237–2256 of human βIV spectrin	Gutzmann et al. (2014), Thome et al. (2014), Schlüter et al. (2017)
mCherry (ms), 632543	1:500	Living Colors^®^, Clontech, Hamburg, Germany	AB_2307319
panNaV (ms), S8809, clone K58/35	1:500	Sigma, St. Louis, MO, USA	Gottlieb and Keller (1997), Gutzmann et al. (2014), Thome et al. (2014)
map2 (gp), 188 004	1:500	Synaptic Systems, Göttingen, Germany	Gumy et al. (2017)
gt anti rb ALEXA, 488, A-11008	1:1000	Molecular Probes, Karlsruhe, Germany	AB_143165
gt anti ms (biotinylated), P0447	1:500	DAKO Agilent, Glostrup, Denmark	AB_2617137
Streptavidin 594, S11227	1:1000	Thermo Scientific, Waltham, MA, USA	AB_2619631

*Rb, rabbit; ms, mouse; gt, goat; gp, guinea pig*.

### AIS Morphological Phenotypes in Mouse Visual Cortex *in Vivo*

To assess AIS phenotypes *in vivo*, perfusion-fixed brains from reporter mice for pyramidal neurons (B6.Cg-Tg(Thy1-GFP)16Jrs/J (Feng et al., 2000), kind gift from Tina Sackmann and Andreas Draguhn, Institute of Physiology and Pathophysiology, Heidelberg University) and interneurons (PVcre/Rosa tomato) were obtained for immunohistochemical analysis (PVcre/Rosa tomato line was a kind gift from Mirko Witte and Jochen Staiger, Institute of Anatomy, University of Göttingen, Germany). All animal procedures were carried out in accordance with the recommendations of the Animal Research Council of Medical Faculty Mannheim, Heidelberg University, and University of Göttingen, respectively. All procedures were approved by the Animal Research Boards of the States of Northrhine-Westfalia and Lower Saxony, respectively. After deep anesthesia with ketamine (120 mg/kg/xylazine (16 mg/kg)), animals were transcardially perfused with 0.9% saline, followed by 1% and 4% phosphate-buffered paraformaldehyde (PFA, pH 7.4), respectively. Brains were removed, cryoprotected in 10% at 4°C sucrose overnight, followed by 30% sucrose for 48 h, and cut on a cryotome at 25 μm. The sections were processed for immunofluorescence as previously described (Gutzmann et al., 2014; Schlüter et al., 2017). Briefly, brains were trimmed to a block including visual cortex and embedded in Tissue Tek^®^ (Sakura Finetek). Double and triple immunofluorescence was performed directly on slides using a fish skin gelatine blocking buffer for all antibodies (0.1% fish skin gelatine (Sigma, Hamburg, Germany), 1% BSA, 0.1% Triton X-100 in PBS).

### Preparation, Transfection and Staining of Rat Visual Cortex Organotypic Cultures

All animal protocols were approved by the Ruhr-University Animal Research Board and the State of Northrhine-Westfalia and Baden-Württemberg. Pigmented Long-Evans rats were used to prepare organotypic tissue cultures (OTCs) of the visual cortex as described previously (Hamad et al., 2014). Briefly, rats were decapitated at the day of birth (P0/P1) and brains were explanted. Blocks from visual cortex were cut into 350 μm slices using a McIlwain tissue chopper (Ted Pella, Redding, CA, USA). Slices were placed on coverslips with a plasma/thrombine coagulate. Cultures were kept in medium consisting of 25% adult horse serum, 25% HBSS, 50% Eagle’s Basal Medium, 1 mM L-Glutamine (all from Life Technologies, Karlsruhe, Germany), and 0.65% D-Glucose (Merck, Darmstadt, Germany). Medium was exchanged two times a week. At the second day *in vitro* (DIV 2), 10 μl of a solution containing 1 mM of uridine, cytosine-ß-D-arabino-furanosid and 5-fluordeoxyuridine (each stock 1 mM, all from Sigma) was added for 24 h to inhibit glial growth. OTCs were transfected at DIV 10 and fixed for immunostaining at DIV 20.

To achieve visualization of complete neuronal morphology, OTCs were transfected with mCherry (under the CMV-promoter, Clontech, Hamburg, Germany) as described (Wirth et al., 2003; Hamad et al., 2011). Briefly, gold particles (Biorad, Munich, Germany) were coated with plasmid DNA encoding mCherry (pmCherry-N1, cat# 632523; Clontech, Heidelberg, Germany) and transfection was carried out at DIV 10 using a hand-held Helios Gene Gun (Bio-Rad, Munich, Germany) with 130 psi helium blast pressure. Subsequently, OTCs were cultured for an additional 10 days before processing for further analysis. A total of 48 OTCs derived from three different preparations (each of six pups) were used in this study.

At DIV 20, OTCs were fixed with prewarmed 4% PFA for 2 h. OTCs were blocked with 3% bovine serum albumin, 3% normal goat serum, and 0.5% Triton X-100 in TBS. Primary antibodies (Table 1) were incubated overnight at 4°C. After several washing steps with 1× TBS, secondary antibodies were applied (for 60 min each). After incubation, OTCs were rinsed several times with 1× TBS and finally switched to PBS. Cultures were mounted on glass coverslips using Roti^®^–Mount (Carl Roth, Karlsruhe, Germany) and sealed with nail polish. To test for antibody specificity, primary antibodies were omitted in control experiments, which completely abolished all stainings.

After confocal assessment was completed, selected cultures were de-coverslipped, rehydrated and incubated in PBS and PBS-Tween-20 (0.05%) for 48 h to elute antibodies. Cultures were blocked with TBS-BSA, and re-incubated in mouse anti mCherry antibody overnight followed by biotinylated goat anti mouse for 3 h, followed by ABC reagent for 2 h (Vector Laboratories Inc., Burlingame, CA, USA, RRID:AB_2336827), and reacted with 3,3′-diaminobenzidine (Sigma) and H_2_O_2_. The reaction product was enhanced with OsO_4_ (Sigma). Cultures were dehydrated and coverslipped in DPX (Sigma). To demonstrate the major cell types, selected neurons and their axonal fields were reconstructed manually at 1000× (Neurolucida, MicroBrightField, Inc., Williston, VT, USA).

OTC for assessment of AIS development were prepared as described above at P0/P1, but cultured on filters (Stoppini et al., 1991). Culture conditions and staining procedures were identical to the roller tube cultures as outlined in the previous section and different culture preparations did not lead to differences in data obtained. Cultures were maintained until DIV 3, 7, 15, 21 and 35.

### Primary Cultures of Cortical Neurons

Cortices of embryonic day 18.5 mice (bl6/C57) were removed and dissected in ice-cold HBSS substituted with 25 mM glucose (Thermo Fisher, Dreieich, Germany). Tissue was further dissociated by enzymatic digestion in Accutase (Thermo Fisher) for 10 min at room temperature and then triturated with a fire-polished Pasteur pipette. Cells were plated on coverslips coated with poly-L-lysine (30 μg/ml) and laminin (2 μg/ml) at a density of 75,000/well for immunofluorescence at DIV 7. Cultures were maintained in Neurobasal medium supplemented with B27, 0.5 mM glutamine, 12.5 μM glutamate and 0.5% penicillin/streptomycin (all compounds from Gibco). For immunofluorescence, cells were fixed in 2% PFA for 10 min, washed, and blocked in fish skin gelatine buffer (see “AIS Morphological Phenotypes in Mouse Visual Cortex *in Vivo*” section) before primary antibody incubation overnight.

### Image Acquisition and Criteria for AIS Onset

A Zeiss AxioImager microscope was used to obtain low magnification survey maps of transfected OTC, highlighting the specific location of all mCherry-positive neurons of each OTC so that cells could later be matched with their original image files. Confocal imaging of transfected and immunostained OTCs was carried out on a Leica SP5 MP using the Leica HC PL APO 63×/1.30 NA Glycerin matched objective and a C1 Nikon confocal microscope with a 60× objective (oil immersion, 1.4 NA), respectively. Scans of mCherry and Alexa 488 signals were acquired sequentially to clearly separate two channels (laser lines: 561 nm and 488 nm, respectively). Z-stacks of various sizes, dependent on OTC thickness between 3 μm and 18 μm, were spaced by 0.5 μm. Image size was 1024 × 1024 pixels (pixel dwell time 720 ns, two frames average), stitched into tiles to cover the entirety of the neuron of interest, and thick optical sections were merged to a maximum intensity projection and saved as tiff and jpg formats. Automatically tiled images were analyzed using Fiji/ImageJ (Rasband, 1997–2012) and enhanced for brightness and contrast in Photoshop CS4 when processed for figure preparation.

Only neurons with a clearly detectable AIS (overlapping signal of mCherry and ßIV-spectrin) were used for further investigation. Soma borders were defined manually. Depending on where the AIS (the ßIV-spectrin positive domain) began, cells were classified into three groups: group I cells with axons of somatic origin, and with proximal AIS at the axon hillock, group II cells with axons of somatic origin, but with distal AIS and a discernible gap between the start of the AIS and the soma, and group III cells with axons with dendritic origin (axon-carrying dendrite cell, AcD) and an AIS either located directly at the origin of the axon or more distal to that point. Several neurochemically distinct types of interneurons can give rise to more than one axon (Meyer and Wahle, 1988); in these cases, each axon and AIS, respectively, was analyzed separately and values were not averaged.

### Neuron Classification

In OTC, neurons were classified as pyramidal neuron (abbreviated as PYR in all figures) or interneuron (abbreviated as IN in all figures) by the following criteria: location in upper or lower cortical layers, soma shape, dendrite configuration (polarized or multipolar), spine density and the initial axonal branching pattern (Ascoli et al., 2008; Hamad et al., 2014). Neurons with apical dendritic polarity, variable numbers of shorter basal dendrites, high spine density, and a descending primary axon with a few obliquely ascending fine collaterals with gracile boutons were classified as pyramidal neurons of supragranular and infragranular layers (Wirth et al., 2003; Hamad et al., 2014). Supragranular pyramidal cells have the soma in the outer 1/3 of the culture and an apical dendrite reaching into or coming close to layer I. Infragranular pyramidal cells have the soma in the lower 2/3 of the cultures and apical dendrites end in middle layers without reaching layer I. We also included pyramidal cells of the layer VIb/subplate with horizontal or oblique orientation in this group. Large cortico-midbrain projecting layer V pyramidal cells with prominent apical tufts in layer I are rarely transfected, because they are less frequent than those with local or callosal axons, and none are in the current sample. Supplementary Figure S1 gives representative examples of the major cell types assessed in the present study; Supplementary Table S1 summarizes other relevant cellular parameters of these representative cells such as soma area, axon length, number of nodes, length of dendrites, number of segments and branch order of the individual dendrites.

A few heavily spiny non-polarized neurons with >6 primary dendrites from middle layers were classified as spiny stellates and were not considered for analysis. Neurons with multipolar or polarized dendritic fields with dendrites being more varicose than those of pyramidal cells, less spiny or rather smooth, and with axons initially branching more profusely within the cell’s dendritic field were classified as non-pyramidal IN.

In about 40% of the non-pyramidal neurons, the axon was sufficiently well stained to classify the type based on the axonal pattern. Neurons giving rise to a local plexus that may extend horizontally and with larger boutons of irregular size forming short terminal elements around neighboring somata suggestive of perisomatic endings were classified as basket cells (BC), many of which are fast-spiking (FS; Ascoli et al., 2008; DeFelipe et al., 2013; Jiang et al., 2015). We therefore grouped large BCs with longer horizontal collaterals and smaller BCs with more restricted axonal fields together. Neurons giving rise to vertical projections either by frequent recurrent branching and thin collaterals with fine boutons not entering layer I were classified as bitufted neurons (BT). Neurons giving rise to an ascending axon branching into an ascending bundle of collaterals reaching into layer I where they adopt a horizontal trajectory were classified as Martinotti cells (MC). BT and MC both target the dendrites of pyramidal neurons and are non-FS cells (Wang et al., 2004; Ascoli et al., 2008; DeFelipe et al., 2013; Jiang et al., 2015). We therefore pooled the BT and MC together as dendrite-targeting neurons. Chandelier cells were not identified in the current samples, likely because they appear in far fewer numbers compared to BCs, which comprise about half of all cortical interneurons, and because they are known to mature later than other interneurons types in various mammalian species (Meyer and Ferres-Torres, 1984; Cruz et al., 2003). Likewise, we could not safely identify bipolar cells, which are rarely transfected because they have very small somata.

### Morphological Analysis of the AIS in OTC

AIS length was determined using a previously described self-written macro (Gutzmann et al., 2014; Schlüter et al., 2017). We used the standard settings of the program to pre-process images by histogram stretching and a sharpening filter to enhance edges. AIS were traced with overlapping ends on proximal and distal sections. The macro automatically straightened each line and data was saved in Excel (Microsoft), plotting number of pixels vs. intensity of ßIV-spectrin signal. To determine AIS length, the open source tool Anaconda (Continuum Analytics) containing the Jupyter Notebook App was utilized. The Jupyter Notebook App allows editing and running notebook documents through a web browser. The coding language Python was employed to write an analysis script in one of these notebook documents. This script determines the AIS length of each cell by evaluating the individual Excel files containing the information about ßIV-spectrin signal plotted vs. number of pixels in the following pattern: all values of ßIV-spectrin are combined and the highest value is used to calculate the cut-off for AIS onset and end. The cut-off was set to 30% of the individual maximum intensity. To avoid taking upward outliers into account, the script checks triplets of AIS signal values. The proximal beginning of AIS is set as the first value of a triplet containing only values that are higher than the calculated cut-off. The distal end of the AIS is determined similarly, starting from the last value of the chart. Pixel difference between beginning and ending is computed and converted into length in micrometer based on the microscope’s calibration.

Neurons with a distal AIS or an AcD were additionally analyzed for the length of the gap between soma and AIS-beginning. Single color channel mode in Photoshop CS4 was used to delete the ßIV-spectrin signal of interfering AIS along the gap. The plotting line in Fiji was started at the individually set soma border, drawn over the whole gap and AIS and finished just beyond the distal AIS end. Gap lengths were defined as the distance between soma border and proximal beginning of the AIS. Only neurons with a clearly defined soma border and a traceable dendrite or axon to the first AIS segment were collected for statistical analysis of gap length.

For the axon diameter calculations, we used a novel approach using a self-written GUI application utilizing Python and the open-source modules matplotlib, opencv, python-bioformats, pyqt and numpy. The maximum intensity projection of the AIS to be measured is used to determine the respective intensity curve. Meta data is saved for pixel to physical dimension conversion. For every neighboring two points on the trace, a virtual rectangle with a width of the distance between the two points and a height of 20 pixels is created and overlaid on the image. The corner points of each rectangle are used to resolve the traced AIS using opencv-transformation tools. The multiple rectangles of the trace are then joined horizontally to a rectangular image spanning the length of the entire trace and height of 20 pixels. This image, now containing only the AIS and its surroundings, is used to determine the diameter of the AIS. Start and end of the AIS are calculated by selecting the first/last pixel with intensity over a 30% threshold (normalized on the channels’ maximum). For the calculation of the diameter, a canny edge filter is applied to the image, which is then divided into vertical slices, each with a thickness of 1 pixel. For each vertical slice, the distance between the first lit pixel (that is the upper edge of the AIS) and last lit pixel (lower edge) is measured, resulting in an overlay of computed diameters on the image. The regions where the overlay fitted the AIS and no noise was interfering were selected. Finally, the mean diameter of these regions was calculated.

Three-dimensional projections from stacks were processed first by blind iterative deconvolution (theoretical PSF based on the optical properties of the microscope and the sample, 10× iteration) according to standard procedures in AutoQuant X3 (Media Cybernetics, Rockville, MD, USA). Subsequently, to visualize x-y-z information and dimensions of AIS in neuronal subtypes, deconvolved files were reconstructed (surface) using Imaris 8.1.2 (Bitplane, Zurich).

### Statistical Analysis

Mean values and standard error of the mean (SEM) of AIS length, gap size and diameter were calculated, plotted and analyzed in Sigma Plot 12.5 Software (Systat Software GmbH). *T*-test and Wilcoxon rank-sum test was carried out for comparison of only two groups. Kruskal-Wallis one-way analysis of variance was applied when comparing three or more groups, followed by Dunn’s correction. Error bars indicate SEM; *p* values and number of samples are given in each graph.

## Results

### AIS Phenotypes *in Vivo*

**Figure 1:**
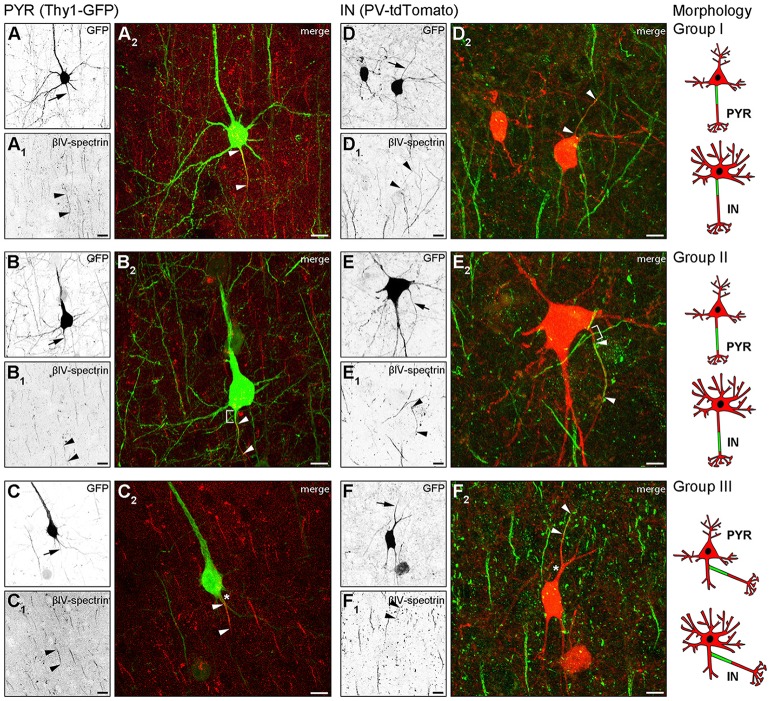
Axon initial segment (AIS) heterogeneity in pyramidal (PYR) neurons and interneurons (IN) *in vivo*. **(A–C)** Layer V PYR neurons in visual cortex sections of adult Thy1-GFP reporter mice, immunostained for βIV-spectrin (red). **(A–A2)** Representative image of a Group I morphology: proximal AIS; arrow in **(A)** shows axon, arrowheads in **(A1+2)** delineate AIS. **(B–B2)** Representative image of a Group II morphology: distal AIS on axon emerging at the soma; arrow in **(B)** shows axon, arrowheads in **(B1+2)** delineate the AIS, bracket in **(B2)** indicates the gap on the axon between soma and AIS onset. **(C–C2)** Representative image of a Group III morphology: axon off a dendrite (AcD), therefore the AIS is located distally from the soma; arrow in **(C)** shows axon, arrowheads in **(C1+2)** delineate AIS, asterisk in **(C2)** indicates dendritic shaft. Scale bars **(A–C)** = 20 μm. **(D–F)** Layer V IN in visual cortex sections of adult PV-tdTomato reporter mice, immunostained for βIV-spectrin (green). **(D–D2)** Representative image of a Group I morphology: proximal AIS; arrow in **(D)** shows axon, arrowheads in **(D1+2)** delineate AIS. **(E–E2)** Representative image of a Group II morphology: distal AIS on axon emerging at the soma; arrow in **(E)** shows axon, arrowheads in **(E1+2)** delineate the AIS, bracket in **(E2)** indicates the gap on the axon between soma and AIS onset. **(F–F2)** Representative image of a Group III morphology: axon off a dendrite (AcD); therefore the AIS is located distally from the soma; arrow in **(F)** shows axon, arrowheads in **(F1+2)** delineate AIS, asterisk in **(F2)** indicates dendritic shaft. Scale bars **(D–F)** = 20 μm, in **(E)** = 15 μm. Cartoons illustrate the three main morphological groups observed.

In Thy1-GFP reporter mice, where a subset of cortical pyramidal neurons is GFP-positive, we found three AIS location phenotypes (Figure 1). In group I cells, the axon emerges from the soma and the AIS locates at the most proximal part of the axon directly at the soma with no discernible gap (Figures 1A–A2, cartoon insert). In group II cells, the axon emerges from the soma, but the AIS is located further distally on the axon with a clearly discernible gap between the first βIV-spectrin immunosignal and the soma (Figures 1B–B2, cartoon insert). The gap is devoid of βIV-spectrin, ankyrinG and voltage-gated sodium channels (panNaV; data not shown). In group III cells, the axon emerges from a dendrite and consequently, the AIS is located far distally from the soma (Figures 1C–C2, cartoon insert).

We next analyzed parvalbumin-tdTomato reporter mice (PV-tdTomato, Figure 1) in which BCs and chandelier cells are labeled (Walker et al., 2016). Parvalbumin (PARV)-expressing interneurons give rise to axons from the soma with AIS residing either proximal directly at the axon hillock (Figures 1D–D2) or distally with a gap to the soma (Figures 1E–E2). We also observed axons off dendrites (Figures 1F–F2), and the AIS can begin directly at the axon origin, or at some distance to the axon origin (Figure 1F2). Consequently, in these cases the distance between AIS and soma is quite substantial. In histological sections, in particular in interneurons, the AIS is either not present nor represented in full extent.

### AIS Development in Rat Visual Cortex OTC

**Figure 2:**
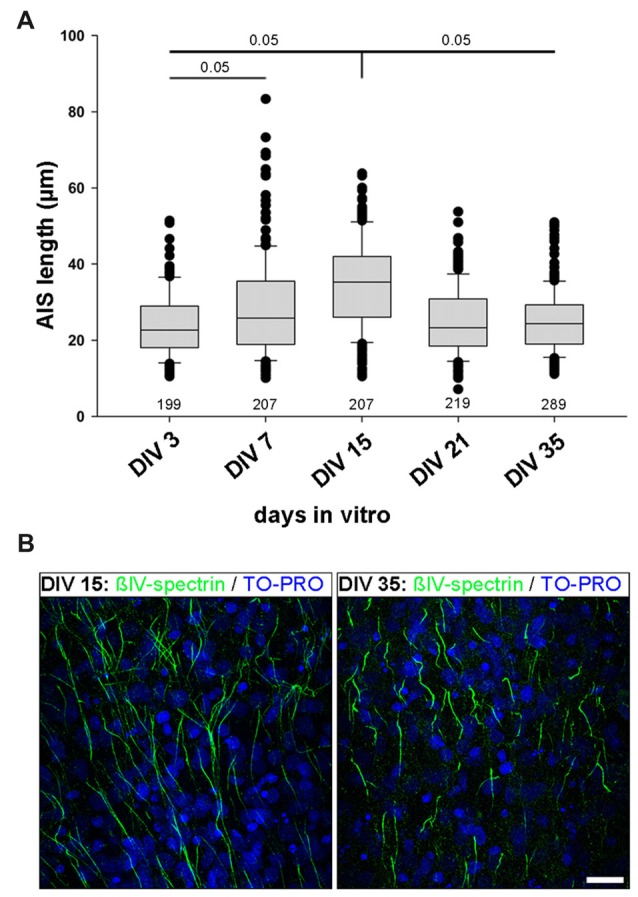
AIS length development in organotypic tissue culture (OTC). **(A)** AIS length measurements at various developmental stages *in vitro* ranging from DIV 3 to DIV 35. Initially AIS elongate until P15, followed by length reduction, which reaches mature states around P35. Note the wider scatter of length distribution already at DIV 7 and overall longer AIS at DIV 15 compared to DIV 35. Kruskal-Wallis one way analysis of variance (ANOVA) followed by *post hoc* Dunn’s correction, *p* value indicated in graph. Length at DIV 15 is significant when compared to all other time points. Length at DIV 3 is significant when compared to DIV 7. Data derived from at least nine cultures per time point from three different preparations. Number of AIS per time point indicated in graph. **(B)** Representative images of AIS lengths in cultures at DIV 15 (left) and DIV 35 (right). AIS are immunostained against βIV-spectrin (green) with nuclear counterstain TO-PRO (blue). Scale bar = 20 μm.

OTC are fully regenerated, all structural elements are contained within the culture, and all neuron types develop in OTC as they do *in vivo*. Accordingly, OTC have long been established as a relevant tool to study structure and function under precisely-to-control conditions (Gähwiler et al., 1997; Wirth et al., 2003; Del Turco and Deller, 2007; Mori et al., 2007; Humpel, 2015). Therefore, we next utilized rat visual cortex-derived OTC to test if the AIS heterogeneity develops in network of cells that are spontaneously active, but lack patterned input from subcortical sources. First, we performed a quantitative analysis of AIS development in OTC in order to determine if OTC undergo a similar AIS maturation as *in vivo* and thus can be seen as equivalent to the *in vivo* situation (Gutzmann et al., 2014). OTC were fixed and stained for AIS scaffolding proteins βIV-spectrin and ankyrinG at 3, 7, 15, 21 and 35 days *in vitro* (DIV). Length measurements were carried out as described previously (Gutzmann et al., 2014). We found that AIS undergo a significant length increase from on average 24.1 μm at DIV 3 to a peak of 34.5 μm at DIV 15 (Figure 2A). Subsequently, AIS shorten to mature levels at DIV 21, which were also seen at DIV35 (Figure 2A). Representative photomicrographs of these different lengths are shown in Figure 2B. This maturational profile largely corresponds to the *in vivo* situation (Gutzmann et al., 2014).

### AIS Phenotypes in Rat Visual Cortex OTC

In total, we analyzed 178 pyramidal neurons classified as 81 supragranular and 85 infragranular as well as 85 non-pyramidal bona fide interneurons. Neurons were sampled from 48 OTC derived from three preparations each from six animals.

In approximately 50% of all interneurons, the quality of the mCherry immunosignal in distal axonal arbors was not optimal, and hence unequivocal subclassification was not possible. These cells were not considered for subtype analysis. From the rest, 16 cells were classified as BC with local or horizontal axon plexus and perisomatic endings, and 24 were dendrite-targeting bitufted and MC (BT/MC).

**Figure 3:**
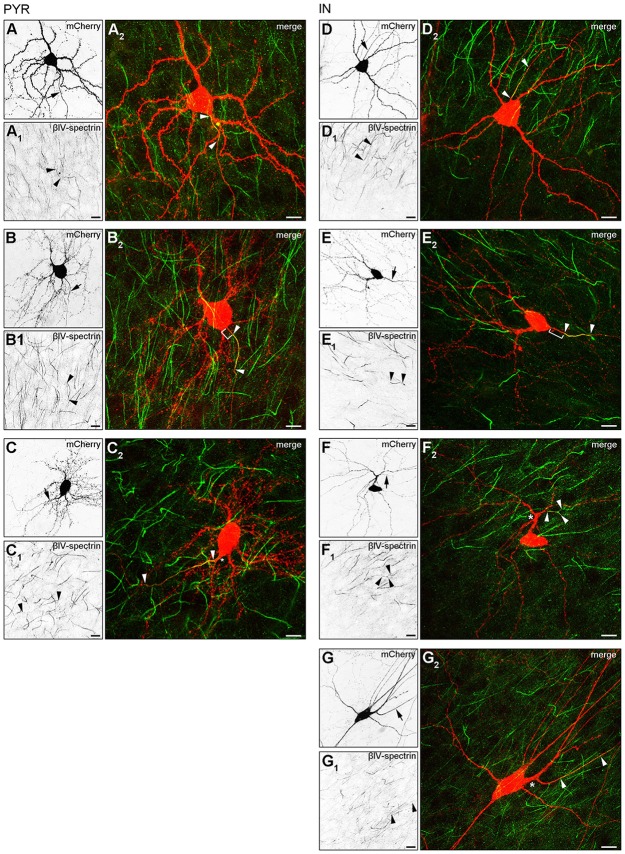
AIS heterogeneity in pyramidal neurons **(A–C)** and interneurons **(D–G)** in OTC. **(A–A2)** Representative image of a pyramidal neuron classified in Group I: proximal AIS; arrow in **(A)** shows axon, arrowheads in **(A1+2)** delineate AIS (mCherry, red; βIV-spectrin, green). The same cell was reconstructed in Imaris to highlight the 3D expansion of the AIS (Supplementary Figure S3A). **(B–B2)** Representative image of a pyramidal neuron classified in Group II: distal AIS on axon emerging at the soma (mCherry, red; βIV-spectrin, green); arrow in **(B)** shows axon, arrowheads in **(B1+2)** delineate the AIS, bracket in **(B2)** indicates gap between AIS and soma. The same cell was reconstructed in Imaris to highlight the 3D expansion of the AIS (Supplementary Figure S3B). **(C–C2)** Representative image of a pyramidal neuron classified in Group III: axon off a dendrite (AcD), therefore the AIS is located distally from the soma; arrow in **(C)** shows axon, arrowheads in **(C1+2)** delineate AIS, asterisk in **(C2)** indicates dendritic shaft.The same cell was reconstructed in Imaris to highlight the 3D expansion of the AIS (Supplementary Figure S3C). Scale bars **(A–C)** = 20 μm. **(D–D2)** Representative image of an interneuron classified in Group I (mCherry, red; βIV-spectrin, green); arrow in **(D)** shows axon, arrowheads in **(D1+2)** delineate the AIS. **(E–E2)** Representative image of an interneuron classified in Group II (mCherry, red; βIV-spectrin, green); arrow in **(E)** shows axon, arrowheads in **(E1+2)** delineate AIS, bracket in **E2** indicates distance from the soma to the onset of the AIS. The same cell was reconstructed in Imaris to highlight the 3D expansion of the AIS (Supplementary Figure S3E). **(F–F2)** Representative image of an interneuron classified in Group III (mCherry, red; βIV-spectrin, green); arrow in **(F)** shows axon, arrowheads in **(F1+2)** delineate AIS with a bifurcation, asterisk in **(F2)** indicates dendritic shaft. The same cell was reconstructed in Imaris to highlight the 3D expansion of the AIS (Supplementary Figure S3F). **(G–G2)** Representative image of an interneuron classified in Group III, with a notably gap between axon onset on dendritic shaft and the beginning of the AIS (mCherry, red; βIV-spectrin, green); arrow in **(G)** shows axon, arrowheads in **(G1+2)** delineate AIS with a clear gap between AIS onset and point of origin of the axon at the dendrite, asterisk in **(G2)** indicates dendritic shaft. Scale bars **(D–G)** = 20 μm.

Our observation of three major AIS location phenotypes from the *in vivo* assessment was confirmed in OTC. Group I (somatic axon with proximal AIS), group II (somatic axon with distal AIS) and group III (dendritic axon origin, AcD, with distal AIS) were found in both pyramidal neurons (Figures 3A–A2,B–B2,C–C2) and interneurons (Figures 3D–D2,E–E2,F–F2,G–G2). Interestingly, we observed two distinct types of AcD cells, especially in interneurons. In one, the AIS starts directly at the point of origin of the axon from the dendrite (Figures 3F–F2), and in the other, the AIS is located distally from the point of origin, adding further to the gap between soma and AIS (Figures 3G–G2). Further, we confirmed that the visible gap between the axon onset and the soma in the AcD phenotype is indeed of dendritic origin as shown by immunostaining against MAP2, panNaV and ankG (Supplementary Figures S2A–A3, B–B3). Using deconvolution and surface reconstruction, we then determined the three-dimensional expansion of AIS in all three morphological classes in pyramidal cells and interneurons (Supplementary Figure S3).

### Interneurons More Often Have Distal AIS Phenotypes than Pyramidal Neurons

**Figure 4:**
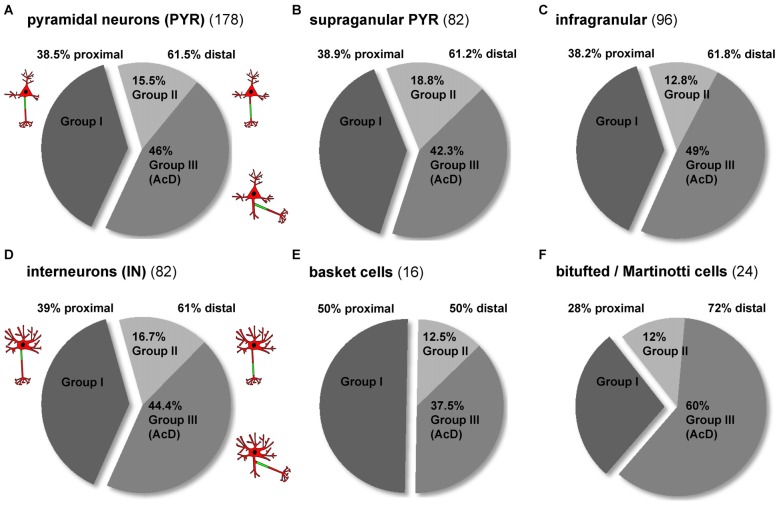
Distribution of AIS location phenotypes among subtypes of pyramidal neurons and interneurons. **(A)** The overall distribution of the three major AIS phenotypes is almost identical in pyramidal neurons and (D) interneurons (dark gray = group I, proximal AIS; light gray = group II, distal AIS, axon with somatic origin; medium gray = group III, axon with dendritic origin). The distribution of distal subtypes (AIS on somatic axon, AIS on dendritc axon (AcD)) favors group III. **(B)** The distribution of AIS phenotypes in supragranular and **(C)** infragranular pyramidal neurons is similar, with a slight trend towards more AcDs in infragranular pyramidal neurons. **(D)** The distribution of AIS phenotypes in interneurons resembles that seen in pyramidal neurons, but the distribution differs for the major subgroups. **(E)** Group I proximal AIS are the dominant phenotype of perisomatically targeting (basket) interneurons, whereas group III AcD are the dominant form (60%) of the **(F)** dendrite-targeting (bitufted and Martinotti) interneurons. Distal AIS on axons with somatic origin occur at an equal proportion (12%) in the two subgroups in **(E,F)**.

When comparing all pyramidal cells to all interneurons, the AIS phenotype groups I, II and III occurred with the same frequency (Figures 4A,D). In both cell classes, about 60% display a distal AIS on an axon with somatic (group II) or dendritic (group III) origin, while somewhat surprisingly, less than 40% of cells fit into the “classical” proximal axon/AIS location group I (Figures 4A,D; PYR 38.5% group I, 15.5% group II, 46% group III; IN 39% group I, 16.7% group II, 44.4% group III). In the cases where a neuron has a distal AIS (regardless of somatic or dendritic axon origin), it is most often an AcD. When looking at pyramidal neurons from supragranular vs. infragranular layers, we observed an almost identical overall distribution of proximal vs. distal AIS, but notably, infragranular neurons more often fall within the AcD classification than supragranular neurons (supragranular: 42.3%; infragranular: 49%; Figures 4B,C). A striking observation was made when comparing the distribution of AIS location phenotypes in interneurons. Here, perisomatic-targeting BCs belong predominantly into group I (50%; Figure 4E). By contrast, dendrite-targeting BT/MCs belong predominantly to group III in that a majority of these interneurons are AcD cells (50% distal AIS, 60% of those group III; Figure 4F).

### Pyramidal Neurons Have Significantly Longer AIS than Interneurons

**Figure 5:**
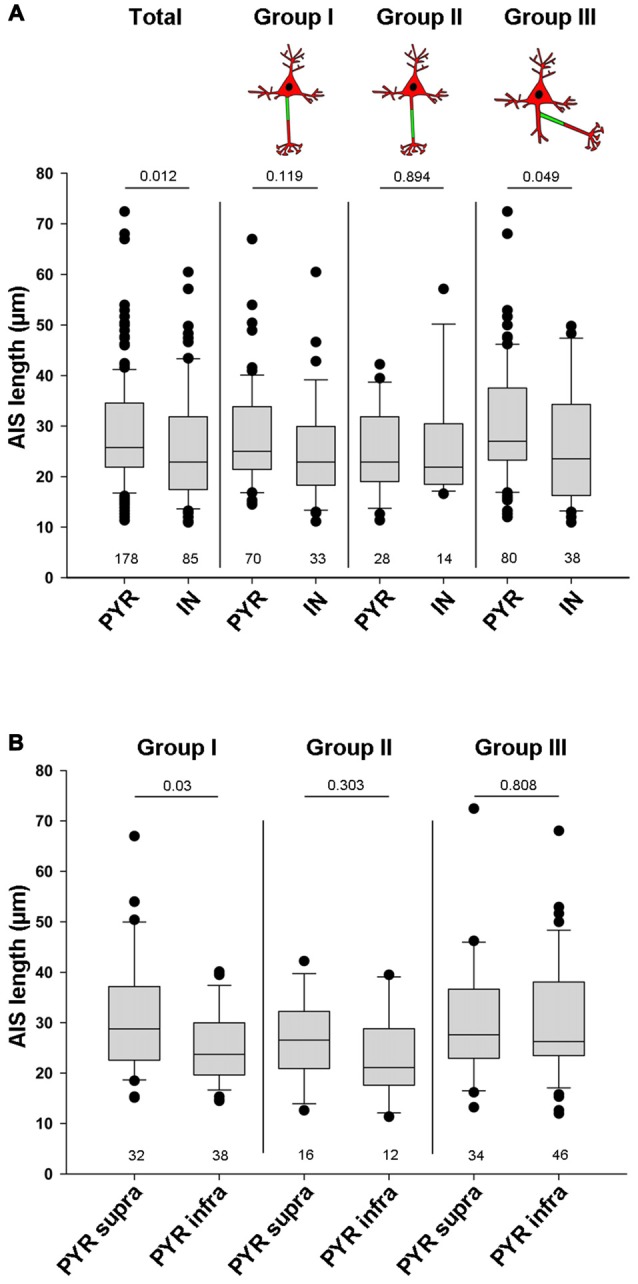
Quantification of overall AIS length in pyramidal and interneuron populations. **(A)** AIS length (in μm) plotted for all pyramidal neurons (PYR) and all interneurons (IN; total, left pair of bars). AIS in PYR are significantly longer than those in IN. No significant differences for AIS length were observed when comparing proximal vs. distal AIS location. However, when comparing AcDs, where the AIS emerges off a basal dendrite, those of PYR are significantly longer. Wilkoxon rank-sum test with *post hoc* correction applying Dunn’s post test, *p* values and *n-numbers* indicated in graph. **(B)** Comparison of the three major AIS phenotypes in supragranular vs. infrgranular pyramidal neurons showed significantly longer AIS in supragranular vs. infragranular neurons. Wilkoxon rank-sum test with *post hoc* correction applying Dunn’s post test, *p* values and *n-numbers* indicated in graph.

Apart from location, another AIS parameter can have significant influence on cellular excitability and function: AIS length. In fact, shortening and elongation are a hallmark feature of developmental and experimentally evoked AIS plasticity *in vivo* and *in vitro* (Cruz et al., 2009; Kuba et al., 2010; Hinman et al., 2013; Gutzmann et al., 2014; Wefelmeyer et al., 2016). When comparing all pyramidal neurons with all interneurons, regardless of AIS location, we found that pyramidal neurons have significantly longer AIS than interneurons (PYR 28.5 μm ± 10.7 vs. IN 25.7 μm ± 11.1; *p* = 0.012; Figure 5A). A similar trend in length difference also occurred when comparing individual neurons classified by AIS group: AIS tended to be shorter in interneurons of group I (PYR: 27.9 ± 10 vs. IN: 25 ± 10.3, *p* = 0.119). No differences were observed for cells in group II (PYR: 25.1 ± 8.7 vs. IN: 26.2 ± 11.6, *p* = 0.894). AIS of group III interneurons again were significantly shorter than those of pyramidal neurons in the same group (PYR: 30.2 ± 11.6 vs. IN: 26 ± 11.7, *p* = 0.049; Figure 5A). When looking at the three major AIS location groups across supragranular and infragranular pyramidal cells, we observed significant length differences for group I only. Here, supragranular neurons have significantly longer AIS than infragranular neurons (supra: 31.1 μm ± 11.9 vs. *infra*: 25.1 μm ± 7.5; *p* = 0.03; Figure 5B), a finding confirming quantitative data from layer 2/3 and layer 5 pyramidal neurons of visual cortex (Gutzmann et al., 2014) and somatosensory cortex (Vascak et al., 2017) *in vivo*. A similar, albeit non-significant trend was observed for group II, where AIS of supragranular pyramidal cells tended to be longer than those of infragranular cells (supra: 26.6 μm ± 8.6 vs. *infra*: 23.1 μm ± 8.8; *p* = 0.303; Figure 5B). No difference was observed for group III (supra: 29.9 μm ± 11.7 vs. *infra*: 30.4 μm ± 11.7; *p* = 0.808; Figure 5B). Within the cohort of classified interneurons, we saw no significant AIS length difference between perisomatic BCs and dendrite-targeting BT/MCs, although a trend for shorter AIS in BCs was apparent (BC: 20.8 μm ± 10.3 vs. BT/MC: 26.4 μm ± 13.7; *p* = 0.136; data not shown).

### The Gap Length between AIS Onset and Soma as well as Axon Diameter Is Similar in Pyramidal Neurons and Interneurons

**Figure 6:**
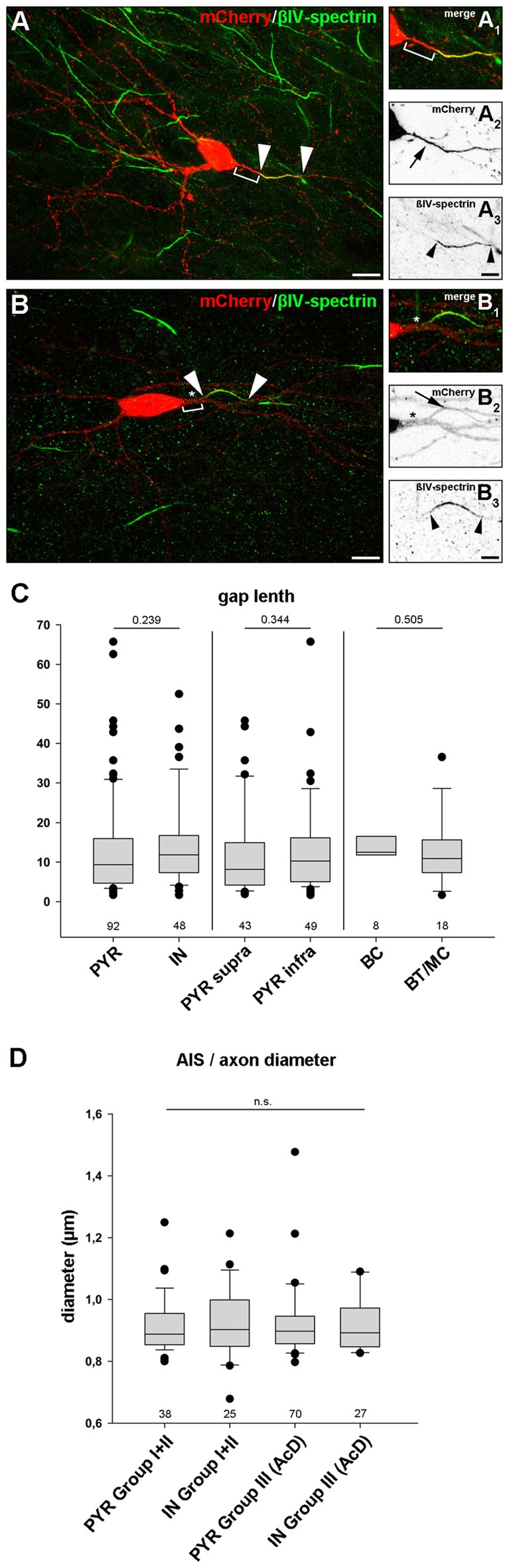
Gap length between AIS and soma is similar in pyramidal neurons and interneurons. **(A–A3)** Representative image of an interneuron classified in group II (distal AIS on somatic axon) to illustrate the gap (bracket in **A+A1**) between soma (mCherry, red; **A–A2**) and proximal AIS (βIV-spectrin, green; **A–A3**). AIS indicated by arrowheads, arrow points to axon. The same cell was reconstructed in Imaris to highlight the 3D expansion of the AIS (Supplementary Figure S3E). **(B–B3)** Representative image of a group III interneuron (AcD). Gap delineated by bracket **(B+B1)**. The dendritic shaft is highlighted by an asterisk. Arrowheads outline the AIS (βIV spectrin, green; **B–B3**) on an axon with dendritic origin (mCherry, red; **B–B2**). **(C)** Gap length was not significantly longer in pyramidal neurons (PYR) compared to interneurons (IN) irrespective of supragranular vs. infragranular location of IN subtype. Pyramidal neurons showed a tendency for a more heterogeneous gap length distribution. Wilkoxon rank-sum test with *post hoc* correction applying Dunn’s post test, *p* values and *n-numbers* indicated in graph. **(D)** Comparison of axon diameter in PYR vs. IN (data pooled for Groups I and II) and AcDs in both cell classes. No significant difference was observed. Wilkoxon rank-sum test with *post hoc* correction applying Dunn’s post test, *p* values and *n-numbers* indicated in graph. Scale bar **(A,B)** = 20 μm, scale bar **(A1–3)** = 10 μm, scale bar **(B1–3)** = 5 μm.

In any cell with a distal AIS, irrespective of whether the axon is of somatic or dendritic origin, the physical gap between the soma and the AIS most likely has significant impact on cellular function. It is hypothesized that even small changes in gap length can have dramatic effects on intrinsic firing properties and/or response times to synaptic input (Gulledge and Bravo, 2016; Hamada et al., 2016). Therefore, we analyzed the length of the gap. It was defined as the distance between the soma and beginning of immunostaining against AIS markers (Figures 6A1–3, Group II; Figures 6B1–3, Group III). We excluded gaps >70 μm from the analysis in order to not skew the distribution towards false positive results, since some cells displayed extremely large gaps of 90–113 μm (see “Noncanonical AIS Morphologies” section). First, we compared all pyramidal neurons with all interneurons, and saw no significant difference in gap length (PYR: 13.5 μm ± 12.6 vs. IN: 14.3 μm ± 10.9, *p* = 0.239; groups II and III were pooled; Figure 6C). No significant difference in gap length was observed when comparing supragranular and infragranular pyramidal neurons (supra: 12.2 μm ± 11.2 vs. *infra*: 13.5 μm ± 11.9, *p* = 0.344). Likewise, no significant difference in gap length between the interneuron classes (perisomatic vs. dendrite-targeting) was seen (BC: 14.4 μm ± 8.8 μm, BT/MC: 12.9 μm ± 8.6 μm, *p* = 0.505). Likewise, axon diameter did not differ significantly between PYR and IN, irrespective of morphological group (PYR non AcD (Groups I + II): 0.92 μm ± 0.02 vs. IN: 0.93 μm ± 0.02; PYR AcD (Group III): 0.92 ± 0.01 vs. IN: 0.92 ± 0.02; Figure 6D).

### Noncanonical AIS Morphologies

**Figure 7:**
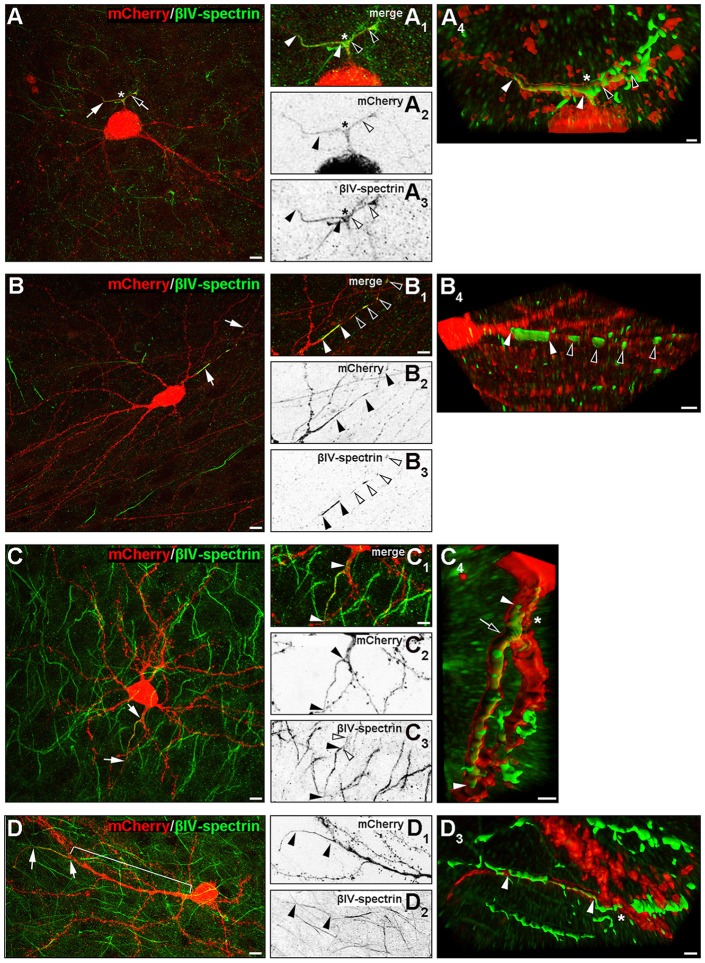
Noncanonical AIS phenotypes in PYR and IN. **(A–A4)** Representative image of an interneuron with a proximal axon bifurcation (asterisk in **A–A4**), along which an AIS is developed, expanding into the two processes after a shared basal shaft (white and black arrows and arrowheads). The length of both AIS is delineated by white and black arrowheads in (**B3**; βIV-spectrin signal). Panel **(A4)** shows the AIS of this cell after deconvolution and surface reconstruction in Imaris to highlight the 3D aspect of the AIS branch point (white and black arrowheads). **(B–B4)** Representative image of βIV-spectrin-positive clusters along the axon of an interneuron (group III, AcD). White arrows in **(B)** indicate the axon, white arrowheads in **(B1)** delineate the length of the primary AIS of this cell (βIV-spectrin, green). Additional immunoreactive clusters termed “extra domains” are outlined by black and white arrowheads, respectively, in **(B1,B3)**. Panel **(B4)** shows the AIS and extra domains of this cell after deconvolution and surface reconstruction in Imaris. **(C–C4)** Representative image of a pyramidal neuron classified in group III (AcD) with a βIV-spectrin-immunoreactive basal dendrite before axon/AIS onset. White arrows in **(C)** and white arrowheads in **(C1)** outline the actual AIS and the proximal “leak” of βIV-spectrin into the basal dendritic shaft (also delineated by white arrowheads in **(C3+4)**, with black arrowheads showing the actual AIS). Panel **(C4)** shows the AIS of this cell after deconvolution and surface reconstruction in Imaris to highlight the actual expansion of βIV-spectrin immunosignal into the dendritic shaft (the proximal beginning of the AIS is highlighted by a black arrow). **(D–D3)** Example of a pyramidal neuron with an extremely large gap between soma and AIS (bracket in **D**). White arrows in **(D)** and black arrowheads in **(D1+2)** delineate the actual AIS. Panel **(D3)** shows the AIS (white arrowheads) at its dendritic origin after deconvolution and surface reconstruction in Imaris. Scale bar **(A–D,D1+2)** = 10 μm, scale bars in **(A1–C3)** for all small inserts = 5 μm, scale bars in 3D reconstruction **(A4)** = 2 μm, **(B4)** = 5 μm, **(C4+D3)** = 3 μm.

Several rather atypical AIS morphologies were observed *in vivo* and in OTC, examples of which are shown in Figure 7 and Supplementary Figures S2C–C3. A number of interneurons showed specific AIS-staining along axonal bifurcations and branch points (Figure 7A). This was not observed in pyramidal neurons. Furthermore, a noteworthy proportion of both pyramidal cells and interneurons exhibited high-intensity spot-like immunoreactivity against βIV-spectrin often at a remarkable distance to the end of the actual AIS, which we tentatively termed “extra domains” (Figure 7B). These occurred in cells that morphologically appeared normal and healthy. Third, we observed what others and we have termed as “leaky” AIS in hippocampal AcD cells (Thome et al., 2014) and dendritic “hot spots” in PARV-positive cells of the olfactory bulb (Kosaka et al., 2008; Thome et al., 2014), showing more or less clearly visible immunoreaction to AIS markers along the basal dendritic shaft and into the emerging axon (Figure 7C, signal delineated by white arrowheads). These qualitative observations were made in a significant proportion of cells, suggesting that these phenotypes unlikely result from culture effects, but rather reflect the significant heterogeneity of AIS in cortical neurons. In fact, extra domains and leaky AIS were seen quite frequently in up to 45% of neurons and merit further investigation. As mentioned above, we also observed several pyramidal neurons and interneurons with extremely long gaps between the proximal AIS border and the axon origin, ranging from 90 μm to 113 μm (Figure 7D). Such extreme cases could result from axonal remodeling after injury induced by lesions during the sectioning process.

## Discussion

### Heterogeneity in Axon Origin

Why is AIS heterogeneity an important subject? A growing number of studies investigating AIS plasticity describe events that indicate an active shifting and/or elongation and shortening of the AIS (reviewed in Adachi et al., 2015; Wefelmeyer et al., 2016; Yamada and Kuba, 2016; Jamann et al., 2017). However, the actual baseline morphology in these systems studies is almost never taken into account. Since a robust AIS live label is currently just emerging (Dumitrescu et al., 2016), the so far reported dynamic range of AIS motion during events of plasticity should be handled with caution. For instance, the shifting and elongating of AIS so far has been observed predominantly in cells where the axon emerges from the soma and the AIS is close to the soma. This configuration is seen in just about one-third of all neurons in the current study. All other neurons where this is not fulfilled have not been analyzed for these forms of structural plasticity as of yet. A fundamental question regarding AIS plasticity is therefore not yet fully answered. Further, the question of whether the various interneuron types undergo comparable forms of plasticity has not been analyzed to date.

Some incoherence exists with regard to the nomenclature of AIS on axons emanating from dendrites, a long known phenomenon in vertebrate neurons, and much more common in non-vertebrates (Triarhou, 2014). We refrained from coining new terms to describe known phenomena, and classified neurons into three distinct axon/AIS groups as outlined in Supplementary Figure S4, using the term AcD cell introduced previously (Thome et al., 2014). The authors showed that roughly 50% of CA1 neurons in mouse hippocampus possess AIS beginning either directly at the point of origin of the axon or with a discernible gap to that point. More recently, Hamada et al. (2016) published experimental and modeling data showing that axon/AIS location should not be viewed isolated from the morphology of the somatodendritic domain. The authors show that in combination with the complexity of the dendritic tree, the location of the axon/AIS is correlated with the somatodendritic capacitance load, and suggest it to be a mechanism for homeostatic scaling of somatic APs (Hamada et al., 2016). The functional consequence of a gap between the AIS and the soma, regardless of whether this gap results from a distal AIS on an axon with somatic origin (Group II) or from a distal AIS/axon emerging off a dendrite (Group III, AcD) is still a matter of debate. It seems logical that the further the AIS is located distally from the soma, the more the AP generation is uncoupled from somatic input. In fact, as shown by recent studies, this results in higher excitability to synaptic input and decreased activation thresholds for AP generation in hippocampal AcD cells (Thome et al., 2014) and cerebellar granule cells of this morphology (Houston et al., 2017). Generally speaking, the optimal AIS length and/or location (that of the most excitability, regardless of whether this is positive or negative for the cell) should be depending on the balance of depolarizing drive and its electrical isolation from the conductance load via the somatodendritic domain (Kuba et al., 2006; Baranauskas et al., 2013). Indeed, a comprehensive modeling study utilizing realistic neurons indicated that there is a physiological optimum for AIS distance to the soma, depending on the cell type and somatodendritic morphology (Gulledge and Bravo, 2016). The authors demonstrate that AIS length and location have different impact on the rheobase: in neurons with a large somatodendritic domain, a longer and more distally located AIS is more optimal, while in smaller neurons, a relatively short and more proximal AIS renders neurons more excitable. Likewise, as shown by Hamada et al. (2016) the gap (measured as branch path length from the soma) is inversely correlated with dendritic tree size and complexity. In fact, “blind” electrophysiological recordings alone gave no indication of which cell type (proximal or distal AIS) was being analyzed (Thome et al., 2014; Hamada et al., 2016), leading to the assumption that AIS position alone cannot determine cellular excitability. So while the distance of the AIS to the soma possibly has direct physiological implications, the corresponding somatodendritic domain seems to be a determining factor as well.

### Methodological Considerations

OTCs are well established culture systems maintaining a 3D environment for long periods *in vitro* to study structure and function under easy-to-control conditions. A majority of processes like development of activity, synaptogenesis, and dendritogenesis occur along the same time line as in cortex *in vivo* (Gähwiler et al., 1997; Wirth et al., 2003; Berghuis et al., 2004; Del Turco and Deller, 2007; Mori et al., 2007; Humpel, 2015). For instance, interneurons develop their typical FS and regular-spiking patterns and connect to regular-spiking pyramidal cells in an adult excitation/inhibition balance by DIV 20 in visual cortex OTC (Klostermann and Wahle, 1999). The activity drives expression of trophic factors like BDNF similar to the *in vivo* situation, with the exception that peak levels evoked by sensory inputs are missing (Gorba and Wahle, 1999; Gorba et al., 1999).

A peculiar observation was the βIV-spectrin positive “extra domains” downstream of the AIS. Such spots could appear as break-down products from AIS or nodes of Ranvier in unhealthy cells, which occur under poor culturing conditions in dissociated neurons and disappear with more optimal culture conditions. However, we now find them in a substantial fraction of cells in DIV 20 OTC long after ontogenetic cell death periods in cultures that are fully recovered and could potentially live on for many further weeks (Humpel, 2015). The corresponding neurons did not display symptoms of degeneration such as nuclear break-down, leakage of mCherry protein, or vacuoles. Although not yet systematically assessed, the distance and periodicity with which the extra domains occur to where AIS scaffolds would normally be found (closer to soma) might imply a more relevant and functional role. One possibility is that these sites represent soon-to-be nodes or preassembled multiprotein “node” packages up for anterograde transport, although their size seems quite large. However, such large microdomains have been observed. In frog spinal cord and cerebral cortex, long nodes of Ranvier of >10 μm in length have been described (Gray, 1970).

### Developmental Aspects and Potential Impact of Species Differences

The developmental profile revealed that overall the AIS of neurons in OTC start out rather short, then elongate to peak values at DIV 15, and shorten until DIV 21 to a final length also seen at DIV 35. Excitability and spontaneous AP firing is initially low and infrequent in OTC, but at DIV 20 neurons display stable membrane potentials, cell type-specific action potential wave forms and spike rates, all of which remain constant in neurons recorded until DIV 140 (Klostermann and Wahle, 1999). Also, in visual cortex of mice *in vivo*, AIS steadily increase in length from shortly after birth until P15 which is around or shortly after eye opening (Gutzmann et al., 2014). Afterwards, AIS undergo a dramatic shortening until the beginning of the critical period of cortical ocular dominance plasticity at P21. By the end of the critical period around P35, AIS have re-elongated to the length which is also observed in the adult cortex, and the variability of individual lengths is much narrower (Gutzmann et al., 2014). A far less dynamic AIS length increase occurred in the non-sensory cingulate cortex, where no influence of visual experience was observed (Gutzmann et al., 2014). Thus, the maturation profile determined here in OTC corresponds in two aspects with the *in vivo* situation, namely the increase to peak lengths until DIV 15 and the wider scatter of individual AIS lengths between DIV 7 and DIV 15. These aspects are either genetically encoded and cell-autonomous or driven by intrinsic cues e.g., depending on the electrical activity developing in OTC. A reason for the wide scatter of length distribution could be the observation in mouse cortex at P8–13 that layer V pyramidal cells display an enormous heterogeneity in firing rate responses and excitability, which seems to be intrinsic to the cells and partly persists into adulthood (Zerlaut et al., 2016). However, layer V in motor cortex harbors four major groups of pyramidal cells distinguished by projection target and their electrophysiological properties (Oswald et al., 2013). This may contribute to the wide scatter of AIS length distribution. It remains to be seen if AIS length and position correlate with the individual functional state of a given neuron, or with target-specificity of a given subset. The previously observed substantial length reduction followed by re-elongation does not occur in OTC, most likely because it is influenced by sensory input (Gutzmann et al., 2014; Schlüter et al., 2017) absent in OTC. Overall, the average length of AIS in rat visual cortex OTC (cultured on filters) is quite similar to the averages determined *in vivo* in mouse visual cortex.

### Functional Consequences of AIS Heterogeneity

The pyramidal cell types in supra- and infragranular layers have clearly different axonal projection targets and projection distances, differ in electrophysiology (Gottlieb and Keller, 1997), in the density of symmetric BC synapses (Fariñas and DeFelipe, 1991; Melchitzky et al., 1997), in mechanisms regulating intrinsic excitability and plasticity (Nataraj et al., 2010), and in the excitatory output to the various types of interneurons (Xu and Callaway, 2009). Yet, they display AIS of similar length, and the AIS phenotypes (Group 1–III) occur in similar proportions, suggesting that these parameters are neither correlated with a cell’s specializations nor constitute a prerequisite for the ability of a cell to undergo plastic changes. This hypothesis is supported by data showing that the onset of an axon either from the soma or a basal dendrite is not driven developmentally by the target region (Hamada et al., 2016).

In mouse visual cortex *in vivo*, AIS of supragranular pyramidal cells are barely longer on average by about 2 μm than those in infragranular layers in mouse visual cortex (Gutzmann et al., 2014). At the structural level, activity-dependent reorganization of the axonal arbors can be elicited, e.g., in the barrel cortex in supragranular cells towards the end of the first postnatal month (Broser et al., 2008). However, AIS length significantly differs between cortical cell classes. Interneurons overall have shorter AIS than pyramidal cells. Whether or not this is genetically or functionally determined has not been addressed yet.

BT/MCs are non-FS neurons (Wang et al., 2004), activated by recurrent pyramidal cell axons, inhibited by interneuron-selective bipolar cells and by BCs (Jiang et al., 2015; Walker et al., 2016), and regulate information processing in pyramidal cell dendrites. They are known to frequently display axons emerging from primary, secondary or even tertiary dendrites (Wahle and Meyer, 1989). These cells being predominantly AcD with distal AIS may be an efficient design principle, because rather few excitatory inputs converging on just the dendrite carrying the axon could trigger the inhibitory output towards the pyramidal cell. Indeed, it was suggested that there is a physiological range of “ideal” AIS distance to the soma; whether or not this accounts for interneurons remains to be determined (Gulledge and Bravo, 2016).

Neurons of all interneuron types may give rise to more than just one axon (Meyer and Wahle, 1988). These axons may arise both from the soma, or both from the same or from different dendrites, or from a soma and a dendrite, and the two axon hillocks can reside close together such that the axon seems to branch within a few micrometer after its point of origin (Meyer and Wahle, 1988). We now show that both axons have an AIS and that their respective lengths are similar. Whether or not the two axons work as independent units on the same cell remains to be determined. The close neighborhood of two axon hillocks on one cell possibly explains some of the noncanonical AIS observed in the present study, namely, βIV-spectrin-immunoreactivity along the entire T-shaped branch point of an axon. However, we also observed βIV-spectrin positive axonal branch points further away from the axon hillock. Multiaxonic cells can be produced *in vitro* by altering proteins essential for neuronal polarity, and these axons are positive for tau and the AIS marker ankyrinG (Muñoz-Llancao et al., 2015). In addition, βIV-spectrin, ankyrinG and voltage-gated sodium channels may be localized in the dendrite which carries the axon. This is another phenomenon previously observed as a “leaky” AIS (Thome et al., 2014). Dendrites carrying an axon are asymmetric in that a fasciculation of microtubules occurs along the side of the dendrite where the axon emerges, whereas the other side of the dendritic cytosol contains microtubulues in the dendrite-typical homogeneous distribution (Peters et al., 1968). However, PARV-positive neurons in the rodent olfactory bulb have been shown to frequently express AIS scaffolding proteins and sodium channels in dendritic “hot spots” 7–50 μm distal from the soma (Kosaka et al., 2008). Interestingly, the membrane undercoating of these particular dendritic segments at the ultrastructural level resembled that of typical AIS (Kosaka et al., 2008).

Perisomatic BCs are feed-forward activated by incoming afferents. Faithful recruitment is enabled by a high density of calcium-permeable AMPA receptors and highly reliable AP firing to deliver feed-forward inhibition, which hyperpolarizes local pyramidal cell groups. BCs containing PARV are FS interneurons and essential for gamma oscillations (Hu et al., 2014). Also in OTC, large BCs respond to very small current injections with strong firing (Klostermann and Wahle, 1999). Interestingly, 50% of BCs had axons originating from the soma and a proximal AIS. AIS location *in vitro* has been recently shown to be regulated by BDNF such that high TrkB signaling moves the AIS proximally towards the soma (Guo et al., 2017). The FS PARV-containing large BCs express mainly TrkB receptors and depend heavily on BDNF derived from pyramidal cells (Gorba and Wahle, 1999; Berghuis et al., 2004; Patz et al., 2004). This suggests that in BCs, a strong and steady BDNF supply stabilizes the AIS close to the soma. It remains to be tested if this “classical” axon/AIS configuration is an essential requirement for FS cell properties.

Why then do we observe just 50% of the BCs with the Group I- phenotype? Likely this is owing to an inhomogeneity of our sample because BCs comprises neurons with very local axons and as well as with long horizontal axonal fields in layers II/III–V, and FS and non-FS types (Battaglia et al., 2013; Jiang et al., 2015; Feldmeyer et al., 2017). Already the PARV-positive BCs are inhomogeneous in mice, with a subset projecting across the corpus callosum (Rock et al., 2017). In the absence of further neurochemical, hodological or electrophysiological markers, we are at this moment unable to divide this group any further. For instance, non-PARV non-FS cholecystokinin-positive BCs employ cannabinoid-1 receptor (CB1R) mediated signaling to enlarge dendritic fields and facilitate synapse development (Berghuis et al., 2004). Thus, AIS location or length may be regulated differently in these cells compared to the BDNF-dependent FS PARV BCs. Interestingly, albeit not residing at the AIS, CB1R signaling influences the AIS during early differentiation in that inhibition of CB1R signaling decreases ankyrinG expression in dissociated hippocampal (mostly pyramidal) neurons, resulting in shorter AIS (Tapia et al., 2017). Presence and strength of such early influences may explain why *in vivo* and *in vitro* as well as in neurons from different species, the overall AIS length varies.

Taken together, these reflections argue for the necessity of a much more detailed analysis of AIS phenotypes in functionally and neurochemically well-delineated neuronal subsets. This future work will then also address the question, how the observed AIS heterogeneity could contribute to neuronal excitability, adaptation and network homeostasis.

## Author Contributions

ME and PW: conceptualization and design of the study. FH, AJ, CR, JM-B, JR, CC, PW and ME: data acquisition and programming. FH, AJ, PW and ME: data analysis. FH, AJ, CS, PW and ME: data interpretation. FH, ME and PW: preparation of manuscript. FH, AJ, CR, JM-B, JR, CC, CS, PW and ME: final approval of the version to be published.

## Conflict of Interest Statement

The authors declare that the research was conducted in the absence of any commercial or financial relationships that could be construed as a potential conflict of interest.

## References

[B1] Adachi R. Yamada R. Kuba H. (2015). Plasticity of the axonal trigger zone. Neuroscientist 21, 255–265. 10.1177/1073858414535986 24847046

[B2] Ascoli G. A. Alonso-Nanclares L. Anderson S. A. Barrionuevo G. Benavides-Piccione R. Burkhalter A.. (2008). Petilla terminology: nomenclature of features of GABAergic interneurons of the cerebral cortex. Nat. Rev. Neurosci. 9, 557–568. 10.1038/nrn2402 18568015 PMC2868386

[B3] Baranauskas G. David Y. Fleidervish I. A. (2013). Spatial mismatch between the Na^+^ flux and spike initiation in axon initial segment. Proc. Natl. Acad. Sci. U S A 110, 4051–4056. 10.1073/pnas.1215125110 23341597 PMC3593864

[B4] Battaglia D. Karagiannis A. Gallopin T. Gutch H. W. Cauli B. (2013). Beyond the frontiers of neuronal types. Front. Neural Circuits 7:13. 10.3389/fncir.2013.00013 23403725 PMC3566547

[B5] Berghuis P. Dobszay M. B. Ibanez R. M. Ernfors P. Harkany T. (2004). Turning the heterogeneous into homogeneous: studies on selectively isolated GABAergic interneuron subsets. Int. J. Dev. Neurosci. 22, 533–543. 10.1016/j.ijdevneu.2004.07.012 15465283

[B6] Broser P. Grinevich V. Osten P. Sakmann B. Wallace D. J. (2008). Critical period plasticity of axonal arbors of layer 2/3 pyramidal neurons in rat somatosensory cortex: layer-specific reduction of projections into deprived cortical columns. Cereb. Cortex 18, 1588–1603. 10.1093/cercor/bhm189 17998276 PMC2430153

[B7] Cajal S. F. R. (1909). Histologie du Système Nerveux de L’homme and des Vertébrés. Paris: Maloine.

[B8] Cruz D. A. Eggan S. M. Lewis D. A. (2003). Postnatal development of pre- and postsynaptic GABA markers at chandelier cell connections with pyramidal neurons in monkey prefrontal cortex. J. Comp. Neurol. 465, 385–400. 10.1002/cne.10833 12966563

[B9] Cruz D. A. Lovallo E. M. Stockton S. Rasband M. Lewis D. A. (2009). Postnatal development of synaptic structure proteins in pyramidal neuron axon initial segments in monkey prefrontal cortex. J. Comp. Neurol. 514, 353–367. 10.1002/cne.22006 19330819 PMC2700028

[B10] Debanne D. Campanac E. Bialowas A. Carlier E. Alcaraz G. (2011). Axon physiology. Physiol. Rev. 91, 555–602. 10.1152/physrev.00048.2009 21527732

[B11] DeFelipe J. López-Cruz P. L. Benavides-Piccione R. Bielza C. Larrañaga P. Anderson S.. (2013). New insights into the classification and nomenclature of cortical GABAergic interneurons. Nat. Rev. Neurosci. 14, 202–216. 10.1038/nrn3444 23385869 PMC3619199

[B12] Del Turco D. Deller T. (2007). Organotypic entorhino-hippocampal slice cultures—a tool to study the molecular and cellular regulation of axonal regeneration and collateral sprouting *in vitro*. Methods Mol. Biol. 399, 55–66. 10.1007/978-1-59745-504-6_5 18309925

[B13] D’Este E. Kamin D. Göttfert F. El-Hady A. Hell S. W. (2015). STED nanoscopy reveals the ubiquity of subcortical cytoskeleton periodicity in living neurons. Cell Rep. 10, 1246–1251. 10.1016/j.celrep.2015.02.007 25732815

[B14] Dumitrescu A. S. Evans M. D. Grubb M. S. (2016). Evaluating tools for live imaging of structural plasticity at the axon initial segment. Front. Cell. Neurosci. 10:268. 10.3389/fncel.2016.00268 27932952 PMC5120105

[B15] Fariñas I. DeFelipe J. (1991). Patterns of synaptic input on corticocortical and corticothalamic cells in the cat visual cortex. II. The axon initial segment. J. Comp. Neurol. 304, 70–77. 10.1002/cne.903040106 2016413

[B16] Feldmeyer D. Qi G. Emmenegger V. Staiger J. F. (2017). Inhibitory interneurons and their circuit motifs in the many layers of the barrel cortex. Neuroscience [Epub ahead of print]. 10.1016/j.neuroscience.2017.05.027 28528964

[B17] Feng G. Mellor R. H. Bernstein M. Keller-Peck C. Nguyen Q. T. Wallace M.. (2000). Imaging neuronal subsets in transgenic mice expressing multiple spectral variants of GFP. Neuron 28, 41–51. 10.1016/s0896-6273(00)00084-2 11086982

[B18] Gähwiler B. H. Capogna M. Debanne D. McKinney R. A. Thompson S. M. (1997). Organotypic slice cultures: a technique has come of age. Trends Neurosci. 20, 471–477. 10.1016/s0166-2236(97)01122-3 9347615

[B19] Gentet L. J. Williams S. R. (2007). Dopamine gates action potential backpropagation in midbrain dopaminergic neurons. J. Neurosci. 27, 1892–1901. 10.1523/JNEUROSCI.5234-06.2007 17314285 PMC6673536

[B21] Gorba T. Klostermann O. Wahle P. (1999). Development of neuronal activity and activity-dependent expression of brain-derived neurotrophic factor mRNA in organotypic cultures of rat visual cortex. Cereb. Cortex 9, 864–877. 10.1093/cercor/9.8.864 10601005

[B20] Gorba T. Wahle P. (1999). Expression of TrkB and TrkC but not BDNF mRNA in neurochemically identified interneurons in rat visual cortex *in vivo* and in organotypic cultures. Eur. J. Neurosci. 11, 1179–1190. 10.1046/j.1460-9568.1999.00551.x 10103114

[B22] Gottlieb J. P. Keller A. (1997). Intrinsic circuitry and physiological properties of pyramidal neurons in rat barrel cortex. Exp. Brain Res. 115, 47–60. 10.1007/pl00005684 9224833

[B23] Gray E. G. (1970). The fine structure of nerve. Comp. Biochem. Physiol. 36, 419–448. 10.1016/0010-406X(70)91021-2 4920890

[B24] Grubb M. S. Shu Y. Kuba H. Rasband M. N. Wimmer V. C. Bender K. J. (2011). Short- and long-term plasticity at the axon initial segment. J. Neurosci. 31, 16049–16055. 10.1523/JNEUROSCI.4064-11.2011 22072655 PMC3232445

[B25] Gulledge A. T. Bravo J. J. (2016). Neuron morphology influences axon initial segment plasticity. eNeuro 3:ENEURO.0085-15.2016. 10.1523/ENEURO.0085-15.2016 27022619 PMC4756267

[B26] Gumy L. F. Katrukha E. A. Grigoriev I. Jaarsma D. Kapitein L. C. Akhmanova A.. (2017). MAP2 defines a pre-axonal filtering zone to regulate KIF1- versus KIF5-dependent cargo transport in sensory neurons. Neuron 94, 347.e7–362.e7. 10.1016/j.neuron.2017.03.046 28426968

[B27] Guo Y. Su Z. J. Chen Y. K. Chai Z. (2017). Brain-derived neurotrophic factor/neurotrophin 3 regulate axon initial segment location and affect neuronal excitability in cultured hippocampal neurons. J. Neurochem. 142, 260–271. 10.1111/jnc.14050 28440877

[B28] Gutzmann A. Ergül N. Grossmann R. Schultz C. Wahle P. Engelhardt M. (2014). A period of structural plasticity at the axon initial segment in developing visual cortex. Front. Neuroanat. 8:11. 10.3389/fnana.2014.00011 24653680 PMC3949221

[B31] Hamada M. S. Goethals S. de Vries S. I. Brette R. Kole M. H. (2016). Covariation of axon initial segment location and dendritic tree normalizes the somatic action potential. Proc. Natl. Acad. Sci. U S A 113, 14841–14846. 10.1073/pnas.1607548113 27930291 PMC5187722

[B29] Hamad M. I. Jack A. Klatt O. Lorkowski M. Strasdeit T. Kott S.. (2014). Type I TARPs promote dendritic growth of early postnatal neocortical pyramidal cells in organotypic cultures. Development 141, 1737–1748. 10.1242/dev.099697 24667327

[B30] Hamad M. I. Ma-Högemeier Z. L. Riedel C. Conrads C. Veitinger T. Habijan T.. (2011). Cell class-specific regulation of neocortical dendrite and spine growth by AMPA receptor splice and editing variants. Development 138, 4301–4313. 10.1242/dev.071076 21865324

[B32] Häusser M. Stuart G. Racca C. Sakmann B. (1995). Axonal initiation and active dendritic propagation of action potentials in substantia nigra neurons. Neuron 15, 637–647. 10.1016/0896-6273(95)90152-3 7546743

[B33] Herde M. K. Iremonger K. J. Constantin S. Herbison A. E. (2013). GnRH neurons elaborate a long-range projection with shared axonal and dendritic functions. J. Neurosci. 33, 12689–12697. 10.1523/JNEUROSCI.0579-13.2013 23904605 PMC6618539

[B34] Hinman J. D. Rasband M. N. Carmichael S. T. (2013). Remodeling of the axon initial segment after focal cortical and white matter stroke. Stroke 44, 182–189. 10.1161/STROKEAHA.112.668749 23233385 PMC3973016

[B35] Houston C. M. Diamanti E. Diamantaki M. Kutsarova E. Cook A. Sultan F.. (2017). Exploring the significance of morphological diversity for cerebellar granule cell excitability. Sci. Rep. 7:46147. 10.1038/srep46147 28406156 PMC5390267

[B36] Hu H. Gan J. Jonas P. (2014). Interneurons. Fast-spiking, parvalbumin^+^ GABAergic interneurons: from cellular design to microcircuit function. Science 345:1255263. 10.1126/science.1255263 25082707

[B37] Humpel C. (2015). Organotypic brain slice cultures: a review. Neuroscience 305, 86–98. 10.1016/j.neuroscience.2015.07.086 26254240 PMC4699268

[B38] Jamann N. Jordan M. Engelhardt M. (2017). Activity-dependent axonal plasticity in sensory systems. Neuroscience [Epub ahead of print]. 10.1016/j.neuroscience.2017.07.035 28739523

[B39] Jiang X. Shen S. Cadwell C. R. Berens P. Sinz F. Ecker A. S.. (2015). Principles of connectivity among morphologically defined cell types in adult neocortex. Science 350:aac9462. 10.1126/science.aac9462 26612957 PMC4809866

[B40] Klostermann O. Wahle P. (1999). Patterns of spontaneous activity and morphology of interneuron types in organotypic cortex and thalamus-cortex cultures. Neuroscience 92, 1243–1259. 10.1016/s0306-4522(99)00009-3 10426481

[B41] Kole M. H. Stuart G. J. (2012). Signal processing in the axon initial segment. Neuron 73, 235–247. 10.1016/j.neuron.2012.01.007 22284179

[B42] Kosaka T. Komada M. Kosaka K. (2008). Sodium channel cluster, βIV-spectrin and ankyrinG positive “hot spots” on dendritic segments of parvalbumin-containing neurons and some other neurons in the mouse and rat main olfactory bulbs. Neurosci. Res. 62, 176–186. 10.1016/j.neures.2008.08.002 18786578

[B43] Kuba H. Ishii T. M. Ohmori H. (2006). Axonal site of spike initiation enhances auditory coincidence detection. Nature 444, 1069–1072. 10.1038/nature05347 17136099

[B44] Kuba H. Oichi Y. Ohmori H. (2010). Presynaptic activity regulates Na^+^ channel distribution at the axon initial segment. Nature 465, 1075–1078. 10.1038/nature09087 20543825

[B45] Leterrier C. (2016). The axon initial segment, 50 years later: a nexus for neuronal organization and function. Curr. Top. Membr. 77, 185–233. 10.1016/bs.ctm.2015.10.005 26781833

[B46] Lorincz A. Nusser Z. (2010). Molecular identity of dendritic voltage-gated sodium channels. Science 328, 906–909. 10.1126/science.1187958 20466935 PMC3546315

[B47] Martina M. Vida I. Jonas P. (2000). Distal initiation and active propagation of action potentials in interneuron dendrites. Science 287, 295–300. 10.1126/science.287.5451.295 10634782

[B48] Melchitzky D. S. Sesack S. R. Lewis D. A. (1997). Axosomatic input to subpopulations of cortically projecting pyramidal neurons in primate prefrontal cortex. Synapse 25, 326–334. 10.1002/(sici)1098-2396(199704)25:4<326::aid-syn3>3.3.co;2-3 9097391

[B49] Meyer G. (1983). Axonal patterns and topography of short-axon neurons in visual areas 17, 18, and 19 of the cat. J. Comp. Neurol. 220, 405–438. 10.1002/cne.902200405 6643736

[B50] Meyer G. Ferres-Torres R. (1984). Postnatal maturation of nonpyramidal neurons in the visual cortex of the cat. J. Comp. Neurol. 228, 226–244. 10.1002/cne.902280209 6480914

[B51] Meyer G. Wahle P. (1988). Early postnatal development of cholecystokinin-immunoreactive structures in the visual cortex of the cat. J. Comp. Neurol. 276, 360–386. 10.1002/cne.902760304 3192767

[B52] Mori M. Gähwiler B. H. Gerber U. (2007). Recruitment of an inhibitory hippocampal network after bursting in a single granule cell. Proc. Natl. Acad. Sci. U S A 104, 7640–7645. 10.1073/pnas.0702164104 17438288 PMC1863441

[B53] Muñoz-Llancao P. Henríquez D. R. Wilson C. Bodaleo F. Boddeke E. W. Lezoualc’h F.. (2015). Exchange protein directly activated by cAMP (EPAC) regulates neuronal polarization through Rap1B. J. Neurosci. 35, 11315–11329. 10.1523/JNEUROSCI.3645-14.2015 26269639 PMC6605123

[B54] Nataraj K. Le Roux N. Nahmani M. Lefort S. Turrigiano G. (2010). Visual deprivation suppresses L5 pyramidal neuron excitability by preventing the induction of intrinsic plasticity. Neuron 68, 750–762. 10.1016/j.neuron.2010.09.033 21092863 PMC2990987

[B55] Oswald M. J. Tantirigama M. L. Sonntag I. Hughes S. M. Empson R. M. (2013). Diversity of layer 5 projection neurons in the mouse motor cortex. Front. Cell. Neurosci. 7:174. 10.3389/fncel.2013.00174 24137110 PMC3797544

[B56] Patz S. Grabert J. Gorba T. Wirth M. J. Wahle P. (2004). Parvalbumin expression in visual cortical interneurons depends on neuronal activity and TrkB ligands during an Early period of postnatal development. Cereb. Cortex 14, 342–351. 10.1093/cercor/bhg132 14754872

[B57] Peters A. Kimerer L. M. (1981). Bipolar neurons in rat visual cortex: a combined Golgi-electron microscope study. J. Neurocytol. 10, 921–946. 10.1007/bf01258522 7031194

[B58] Peters A. Proskauer C. C. Kaiserman-Abramof I. R. (1968). The small pyramidal neuron of the rat cerebral cortex. The axon hillock and initial segment. J. Cell Biol. 39, 604–619. 10.1083/jcb.39.3.604 5699934 PMC2107556

[B59] Rasband M. N. (2010). The axon initial segment and the maintenance of neuronal polarity. Nat. Rev. Neurosci. 11, 552–562. 10.1038/nrn2852 20631711

[B60] Rasband W. S. (1997–2012). ImageJ. National Institutes of Health, Bethesda, MD, USA. Available online at: https://imagej.nih.gov/ij/

[B61] Rock C. Zurita H. Lebby S. Wilson C. J. Apicella A. J. (2017). Cortical circuits of callosal gabaergic neurons. Cereb. Cortex [Epub ahead of print]. 10.1093/cercor/bhx025 28174907

[B62] Schlüter A. Del Turco D. Deller T. Gutzmann A. Schultz C. Engelhardt M. (2017). Structural plasticity of synaptopodin in the axon initial segment during visual cortex development. Cereb. Cortex 27, 4662–4675. 10.1093/cercor/bhx208 28922860

[B63] Sloper J. J. Powell T. P. (1979). A study of the axon initial segment and proximal axon of neurons in the primate motor and somatic sensory cortices. Philos. Trans. R. Soc. Lond. B Biol. Sci. 285, 173–197. 10.1098/rstb.1979.0004 88058

[B64] Stoppini L. Buchs P. A. Muller D. (1991). A simple method for organotypic cultures of nervous tissue. J. Neurosci. Methods 37, 173–182. 10.1016/0165-0270(91)90128-m 1715499

[B65] Tapia M. Dominguez A. Zhang W. Del Puerto A. Ciorraga M. Benitez M. J.. (2017). Cannabinoid receptors modulate neuronal morphology and ankyring density at the axon initial segment. Front. Cell. Neurosci. 11:5. 10.3389/fncel.2017.00005 28179879 PMC5263140

[B66] Thome C. Kelly T. Yanez A. Schultz C. Engelhardt M. Cambridge S. B.. (2014). Axon-carrying dendrites convey privileged synaptic input in hippocampal neurons. Neuron 83, 1418–1430. 10.1016/j.neuron.2014.08.013 25199704

[B67] Triarhou L. C. (2014). Axons emanating from dendrites: phylogenetic repercussions with Cajalian hues. Front. Neuroanat. 8:133. 10.3389/fnana.2014.00133 25477788 PMC4235383

[B68] Vascak M. Sun J. Bear M. Jacobs K. M. Povlishock J. T. (2017). Mild traumatic brain injury evokes pyramidal neuron axon initial segment plasticity and diffuse presynaptic inhibitory terminal loss. Front. Cell. Neurosci. 11:157. 10.3389/fncel.2017.00157 28634442 PMC5459898

[B69] Wahle P. Meyer G. (1989). Early postnatal development of vasoactive intestinal polypeptide- and peptide histidine isoleucine-immunoreactive structures in the cat visual cortex. J. Comp. Neurol. 282, 215–248. 10.1002/cne.902820206 2708596

[B70] Walker F. Möck M. Feyerabend M. Guy J. Wagener R. J. Schubert D.. (2016). Parvalbumin- and vasoactive intestinal polypeptide-expressing neocortical interneurons impose differential inhibition on Martinotti cells. Nat. Commun. 7:13664. 10.1038/ncomms13664 27897179 PMC5141346

[B71] Wang Y. Toledo-Rodriguez M. Gupta A. Wu C. Silberberg G. Luo J.. (2004). Anatomical, physiological and molecular properties of Martinotti cells in the somatosensory cortex of the juvenile rat. J. Physiol. 561, 65–90. 10.1113/jphysiol.2004.073353 15331670 PMC1665344

[B72] Wefelmeyer W. Puhl C. J. Burrone J. (2016). Homeostatic plasticity of subcellular neuronal structures: from inputs to outputs. Trends Neurosci. 39, 656–667. 10.1016/j.tins.2016.08.004 27637565 PMC5236059

[B73] Wirth M. J. Brun A. Grabert J. Patz S. Wahle P. (2003). Accelerated dendritic development of rat cortical pyramidal cells and interneurons after biolistic transfection with BDNF and NT4/5. Development 130, 5827–5838. 10.1242/dev.00826 14573511

[B74] Xu X. Callaway E. M. (2009). Laminar specificity of functional input to distinct types of inhibitory cortical neurons. J. Neurosci. 29, 70–85. 10.1523/JNEUROSCI.4104-08.2009 19129386 PMC2656387

[B75] Yamada R. Kuba H. (2016). Structural and functional plasticity at the axon initial segment. Front. Cell. Neurosci. 10:250. 10.3389/fncel.2016.00250 27826229 PMC5078684

[B76] Zerlaut Y. Telenczuk B. Deleuze C. Bal T. Ouanounou G. Destexhe A. (2016). Heterogeneous firing rate response of mouse layer V pyramidal neurons in the fluctuation-driven regime. J. Physiol. 594, 3791–3808. 10.1113/JP272317 27146816 PMC4929333

