# Supplementary material for: Heterogeneity of the Axon Initial Segment in Interneurons and Pyramidal Cells of Rodent Visual Cortex
Source: Front Cell Neurosci. 2017 Nov 6;11:332. doi: 10.3389/fncel.2017.00332 (PMC5684645; doi:10.3389/fncel.2017.00332)
Supplement: Supplementary file 1 [file Presentation_1.pdf]

Supplementary Material

Heterogeneity of the Axon Initial Segment in Interneurons and Pyramidal Cells of Rodent Visual Cortex

Felix Höfflin[1°], Alexander Jack[2°], Christian Riedel[2], Julia Mack-Bucher[3+], Johannes Roos[1], Corinna Corcelli[1], Christian Schultz[1], Petra Wahle[2*], Maren Engelhardt[1*]

[1] Institute of Neuroanatomy, Medical Faculty Mannheim, Center for Biomedicine and Medical Technology Mannheim (CBTM), Heidelberg University, Germany

[2] Developmental Neurobiology, Department of Zoology and Neurobiology, Ruhr-University Bochum, Germany

[3] Live Cell Imaging Core Mannheim (LIMA), Medical Faculty Mannheim, CBTM, Heidelberg University, Germany

*** Correspondence:**

Maren Engelhardt

maren.engelhardt@medma.uni-heidelberg.de

Petra Wahle

petra.wahle@rub.de

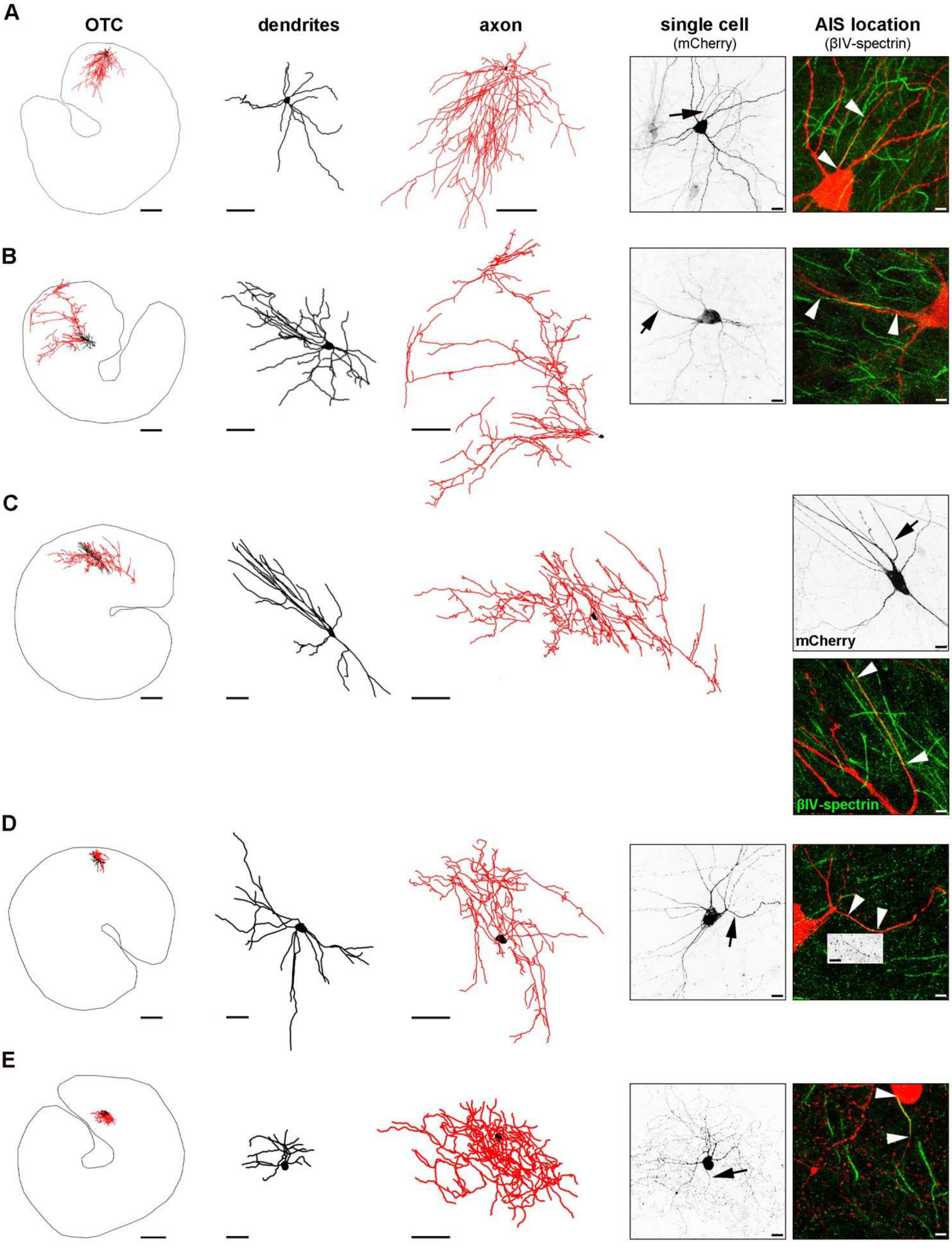
A
OTC
dendrites
axon
single cell
(mCherry)
AIS location
(βIV-spectrin)
B
C
mCherry
βIV-spectrin
D
E

Supplementary Fig. S1 Reconstructions of interneurons representing the major subgroups. From left to right: position of the cell in the OTC; dendrites in black; axon arbor in red; AIS of the neuron (immunofluorescence against mCherry, red and βIV-spectrin, green). Black arrows highlight the axon, white arrowheads indicate the length and location of the AIS. **(A)** Bitufted interneuron with arcade axon forming numerous descending collaterals. **(B)** Martinotti interneuron with ascending collaterals projecting horizontally in layer I. **(C)** Large basket cell with horizontal axon collaterals. **(D)** Small basket cell with local plexus in layer II. Insert in far right panel is an inverted image of the βIV-spectrin immunosignal, which in this particular cell was somewhat weak. **(E)** Small (nest) basket cell with extremely local axonal field. Scale bars A-D = 1 mm for the OTC, 100 µm for dendrites, 200 µm for axons. Scale bars E = 1 mm for the OTC, 50 µm for dendrites, 100µm for axons. Scale bars for mCherry micrographs A-E = 20 µm. Scale bars for βIV-spectrin IF A-E = 5 µm. Scale bar for insert in D = 5 µm.

Supplementary Table 1: Somatodendritic parameters of the reconstructed interneurons shown in Supplementary Figure S1. Almost as expected, neurons with complex axons and large dendrites have larger somata. In two of the three cells with an axonal origin from a dendrite the AcD is the longest and highly branched dendrite, however, in a large basket cell (C), the AcD has an average length and branching. Whether or not interneuronal AcD cells have the most prominent dendrites remains to be tested.

**Cell**	**Soma area**	**Axon length, number of nodes, origin**	**Length, number of segments, and branch order of the individual dendrites**
**A**<br>Arcade	270 µm^2	32.046 µm, 174, somatic	897 µm, 7, 4; 317 µm, 3, 2; 264 µm, 1, 1; 222 µm, 3, 2; 161 µm, 1, 1; 104 µm, 1, 1; 96 µm, 1, 1
**B**<br>Martinotti	552 µm^2	29.509 µm, 240, dendritic	**AcD: 1988 µm, 25 , 7;** 944 µm, 15, 5; 926 µm, 13, 6; 754 µm, 11, 6; 494 µm, 7, 4
**C**<br>Large BC	683 µm^2	28.376 µm, 322, dendritic	**AcD: 2515 µm, 23, 9;** 1327 µm, 11, 5; 876 µm, 7, 3; 418 µm, 7, 4; 374 µm, 1, 1; 249 µm, 1, 1
**D**<br>Small BC	373 µm^2	7.932 µm, 122, dendritic	**AcD: 391 µm, 3, 2;** 574 µm, 5, 3; 671 µm, 11, 5; 360 µm, 7, 3; 137 µm, 1, 1; 138 µm, 1, 1
**E**<br>Nest BC	214 µm^2	9.676 µm, 163, somatic	409 µm, 11, 5; 298 µm, 9, 4; 180 µm, 5, 3; 87 µm, 3, 2

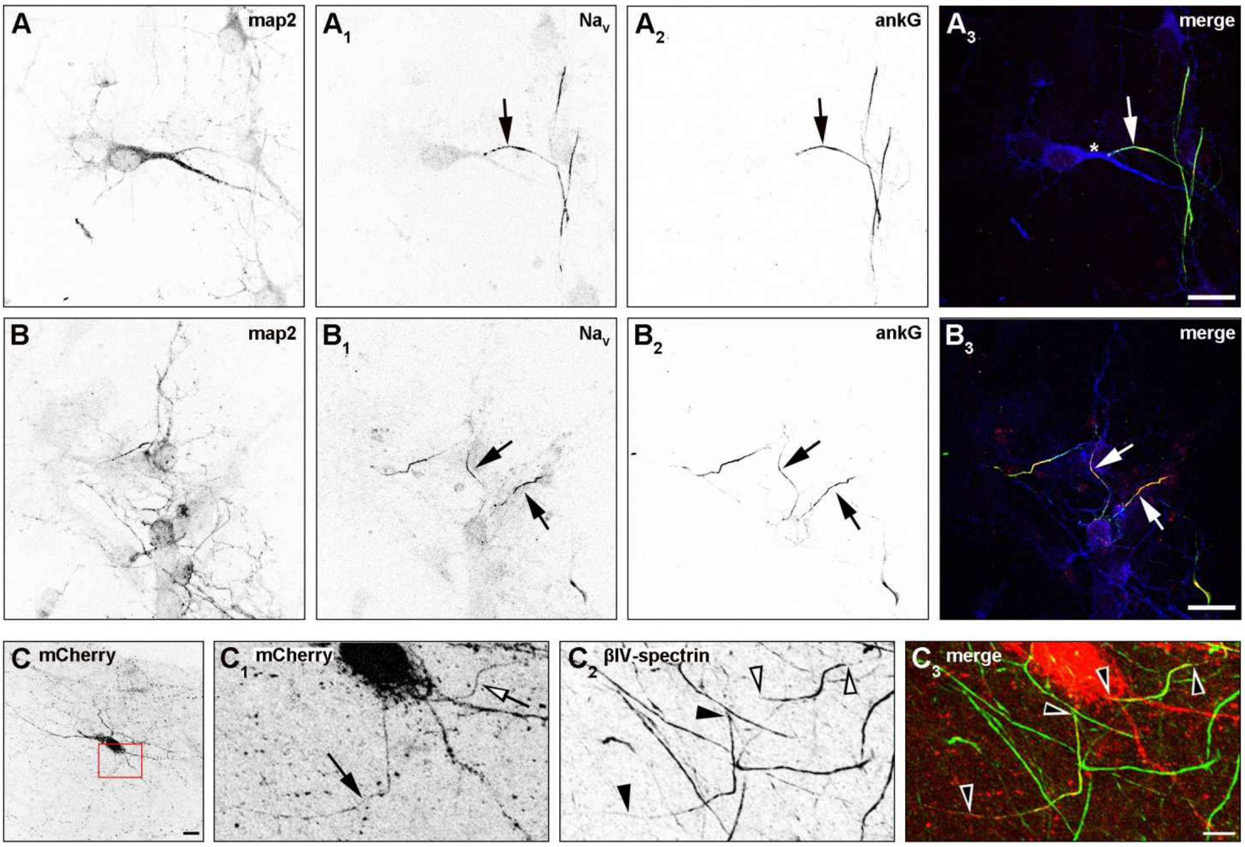

Supplementary Fig. S2. AIS heterogeneity *in vitro* and *ex vivo*. (A-A_3) Representative image of a group III neuron with an axon of dendritic origin and a distal AIS (AcD, arrow) in E18.5 cortex-derived primary neuronal cultures at DIV7. Immunofluorescence against the dendritic marker map2 **(A)**, the voltage-gated sodium channel marker pan NaV **(A_1)**, the AIS scaffolding protein ankG **(A_2)**, and all channels merged **(A_3)**. **(B-B_3)** Representative image of a neuron with two axons and two AIS (arrows), immunostained for map2 **(B)**, pan NaV **(B_1)**, ankG **(B_2)**, and all channels merged **(B_3).** This phenotype was also observed in OTC, albeit at very low frequency (5 out of 260 neurons). **(C)** An interneuron in a DIV 20 OTC with two axons (black and white arrows, **C_1**, **C_2**) and two AIS (black and white arrowheads, **C_2**, **C_3**). Red square in C indicates region at higher magnification in C1-C3. Scale bars A + B = 10 µm; C = 20 µm; C1-3 = 5 µm.

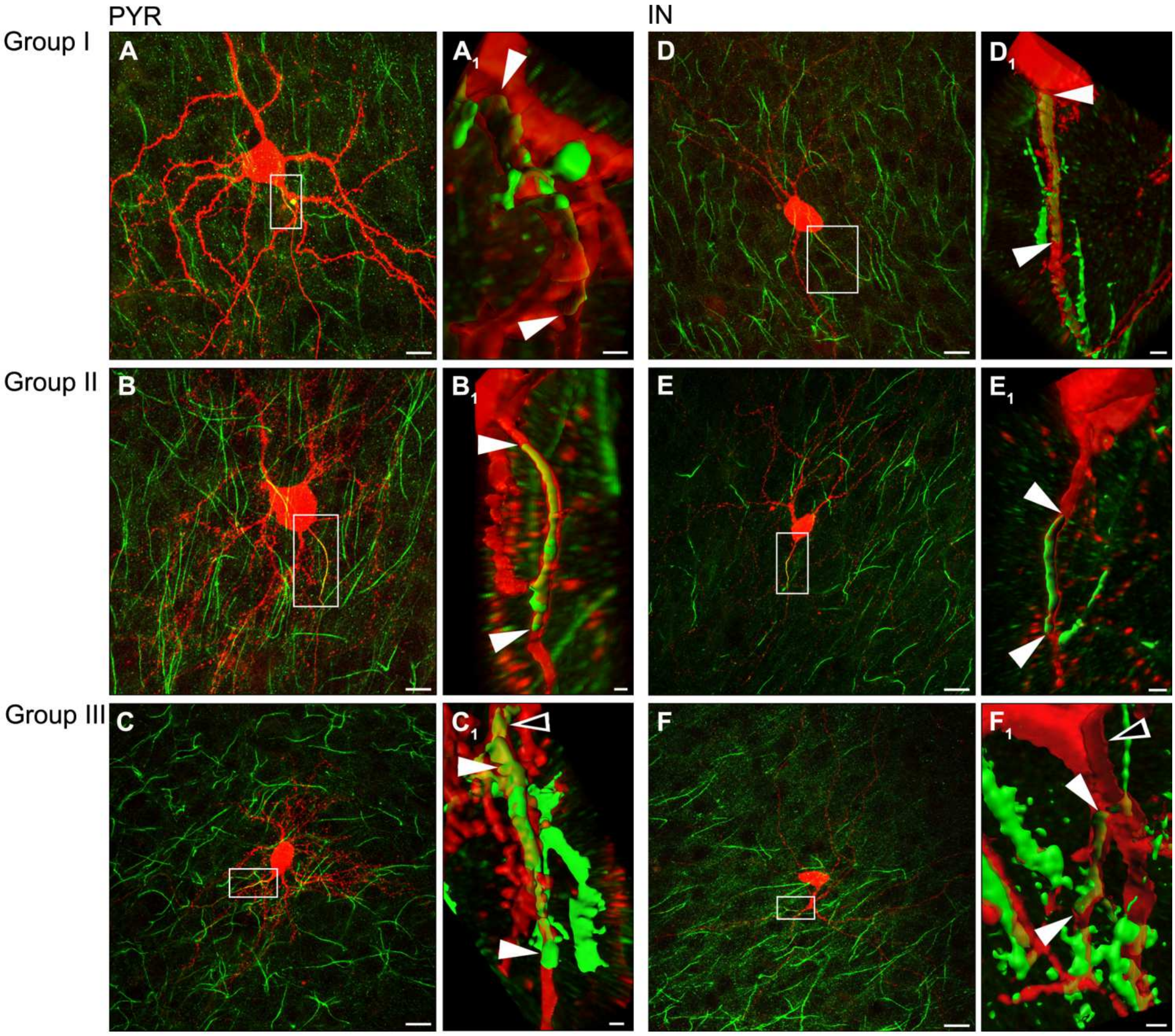

Supplementary Fig. S3. Three dimensional reconstruction of representative PYR (A-C) and IN (D-F) cells from the three morphological groups. (A-A1) A pyramidal neuron (see also Fig. 3A) from Group I: proximal AIS. Magnification and surface reconstruction in Imaris (A1) shows βIV-spectrin immunosignal (green) in a 3D context of the axon (AIS outlined by arrowheads). The image shows a slightly tilted view of the AIS in A (3° to the right). **(B-B1)** A pyramidal neuron (see also Fig. 3B) from Group II: distal AIS on axon emerging at the soma. Magnification and surface reconstruction in Imaris (B1) shows βIV-spectrin immunosignal (green) in a 3D context of the axon (AIS outlined by arrowheads). The gap to the soma is clearly visible. The image shows a slightly tilted view of the AIS in B (5° to the left). **(C-C1)** A pyramidal neuron (see also Fig. 3C) from Group III: axon off a dendrite (AcD). Magnification and surface reconstruction in Imaris (C1) shows βIV-spectrin immunosignal (green) in a 3D context of the axon (AIS outlined by white arrowheads). The dendritic shaft between AIS and soma is highlighted by a black arrowhead. The image shows a tilted view of the AIS in C (35° counterclockwise rotation, then 2° to the right). **(D-D1)** An interneuron classified as bitufted from Group I: proximal AIS. Magnification and surface reconstruction in Imaris (D1) shows βIV-spectrin immunosignal (green) in a 3D context of the axon (AIS outlined by arrowheads). The image shows a slightly tilted view of the AIS in A (25° clockwise rotation, then 2°

to the right). **(E-E1)** An interneuron from Group II: distal AIS on axon emerging at the soma. Magnification and surface reconstruction in Imaris (D1) shows βIV-spectrin immunosignal (green) in a 3D context of the axon (AIS outlined by arrowheads) with the gap to the soma clearly visible. The image shows a slightly tilted view of the AIS in A (1° to the right). **(F-F1)** An interneuron from Group III: axon off a dendrite (AcD). Magnification and surface reconstruction in Imaris (D1) shows βIV-spectrin immunosignal (green) in a 3D context of the axon (AIS outlined by arrowheads). The dendritic origin of the AIS is highlighted by a black arrowhead. The image shows a tilted view of the AIS in C (25° counterclockwise rotation, then 4° to the right). Scale bars A-F = 20 µm; A1, B1, C1 = 2 µm, D1+E1 = 3 µm, F1 = 2 µm.

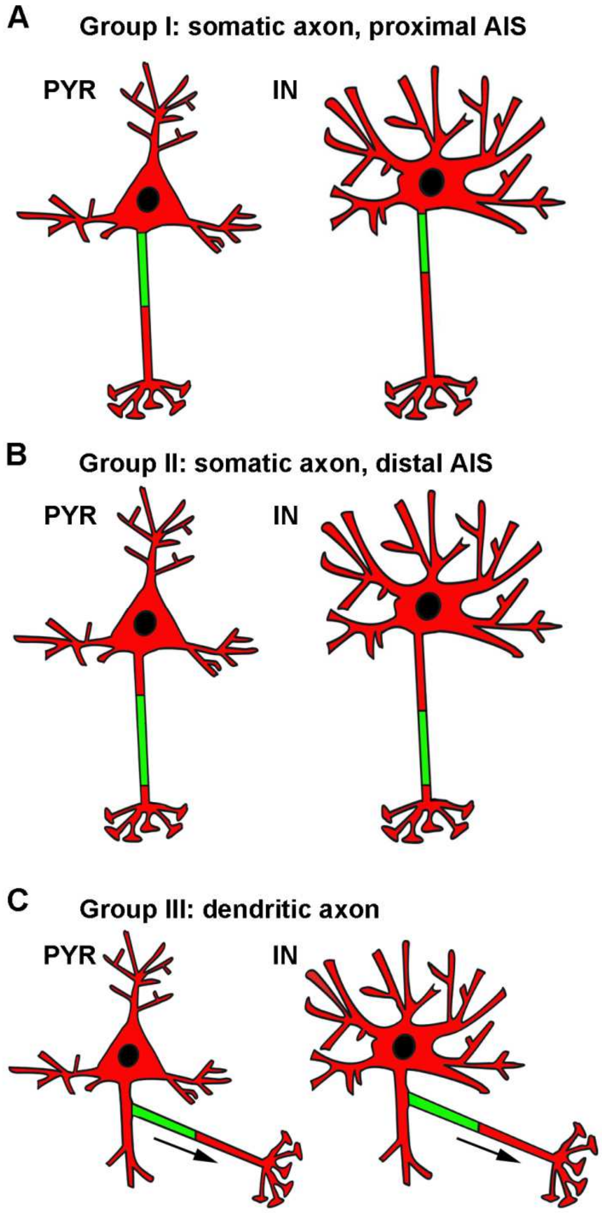

Supplementary Fig. S4 Summary of the major classes of AIS phenotypes in PYR and IN. (A) A large fraction of PYR and IN were classified as group I cells with a somatic axon origin and a proximal AIS location directly at the axon hillock. **(B)** A small fraction of PYR and IN were classified as group II cells with somatic axon origin but a distal AIS location with a discernable gap to the soma. **(C)** A large fraction of PYR and of IN (predominant in dendrite-targeting IN) were classified as group III cells, in which the axon is of dendritic origin (axon-carrying dendrite cells, AcD). Black arrows in C indicate that the proximal AIS border in AcD cells can be either directly at the axon origin or distal to that branch point. In this group, the dendritic segment from which the axon originates represents the “gap”, and since the dendrite may branch before the axons starts, this “gap” to the soma was highly variable in length and in fact quite long, spanning 50 µm or more.
